# Two closely related Rho GTPases, Cdc42 and RacA, of the en-dophytic fungus *Epichloë festucae* have contrasting roles for ROS production and symbiotic infection synchronized with the host plant

**DOI:** 10.1371/journal.ppat.1006840

**Published:** 2018-01-25

**Authors:** Yuka Kayano, Aiko Tanaka, Daigo Takemoto

**Affiliations:** Graduate School of Bioagricultural Sciences, Nagoya University, Chikusa, Nagoya, Japan; University of Nebraska-Lincoln, UNITED STATES

## Abstract

*Epichloë festucae* is an endophytic fungus which systemically colonizes temperate grasses to establish symbiotic associations. Maintaining symptomless infection is a key requirement for endophytes, a feature that distinguishes them from pathogenic fungi. While pathogenic fungi extend their hyphae by tip growth, hyphae of *E*. *festucae* systemically colonize the intercellular space of expanding host leaves via a unique mechanism of hyphal intercalary growth. This study reports that two homologous Rho GTPases, Cdc42 and RacA, have distinctive roles in the regulation of *E*. *festucae* growth *in planta*. Here we highlight the vital role of Cdc42 for intercalary hyphal growth, as well as involvement of RacA in regulation of hyphal network formation, and demonstrate the consequences of mutations in these genes on plant tissue infection. Functions of Cdc42 and RacA are mediated via interactions with BemA and NoxR respectively, which are expected components of the ROS producing NOX complex. Symbiotic defects found in the *racA* mutant were rescued by introduction of a Cdc42 with key amino acids substitutions crucial for RacA function, highlighting the significance of the specific interactions of these GTPases with BemA and NoxR for their functional differentiation in symbiotic infection.

## Author summary

*Epichloë festucae* is a fungus, which grows inside the plant tissue of temperate grasses in order to establish symbiotic associations. In contrast to disease causing fungi, *E*. *festucae* does not trigger a defense response in the host plant. Strict control of the biomass, as well as the growth pattern in host plants are expected to be key requirements for avoiding a defense response by the plant. However, the mechanism by which this symbiotic infection is established, remains largely unknown. In this study, we report that two highly homologous Rho GTPases, Cdc42 and RacA, have distinctive roles in the regulation of the fungus’ growth inside the plant. It was observed that hyphal cells of the fungus are attached to plant cells via the extracellular matrix, and it grows via unique intercalary hyphal extension, rather than via hyphal tip growth, which is normally the case for standard filamentous fungi. *cdc42* mutants were able to colonize younger host tissue, but the hyphae were fragmented when tillers of the host plant elongated, indicating that Cdc42-mediated intercalary hyphal growth is a key event for systemic infection of the fungus in host plants. In contrast, RacA is involved in the establishment of the hyphal network in the intercellular spaces of the host plant, via formation of hyphal cell-cell fusion, which may be required to minimize stress on the host plant caused by the infection.

## Introduction

In nature, many plant species harbor various microbes inside their tissues [1]. Below ground tissues of plants (i.e. the root system) are the major site for plant-microbe interactions, where arbuscular mycorrhiza, ectomycorrhiza and nitrogen fixing bacteria colonize the root system with beneficial consequences on the growth of plants [2]. There are large numbers of endophytic microbes with no obvious benefit for the host plants, making it difficult to distinguish between parasitic or mutualistic interactions [1], as asymptomatic endophytes may protect the plants from pathogenic organisms in certain circumstances, by competing for nutrient acquisition and infection spaces, or by inducing weak defense reactions of host plants.

There are relatively fewer known endophytic microbes in aerial tissues of plants. One representative of aboveground endophytic fungi, is *Epichloë*, which systemically colonizes the intercellular spaces of leaf primordia, leaf sheaths, blades and tillers to establish symbiotic associations with temperate grasses of the subfamily Pooideae [3–5]. The hyphae of *Epichloë* endophytes eventually grow into the inflorescence tissues of reproductive tillers to colonize the embryo of plant seeds, which enable *Epichloë* endophytes to propagate with the host plant. Colonization by *Epichloë* endophytes confers various benefits on host plant growth and fitness to environmental stresses through the production of distinct classes of biologically active metabolites (e.g. alkaloids) for improving plant resistance to a range of biotic and abiotic stresses, including drought, disease and animal/insect herbivores [4, 6–9]. The organization of alkaloid loci and abundant transposon-derived repeat blocks in the genome sequences of *Epichloë* suggest that *Epichloë* endophytes are under selection pressure for the production of diverse alkaloids [10]. This is probably an important feature of *Epichloë* endophytic fungi to enhance fitness of host plants to variable environmental stresses.

**Fig 1:**
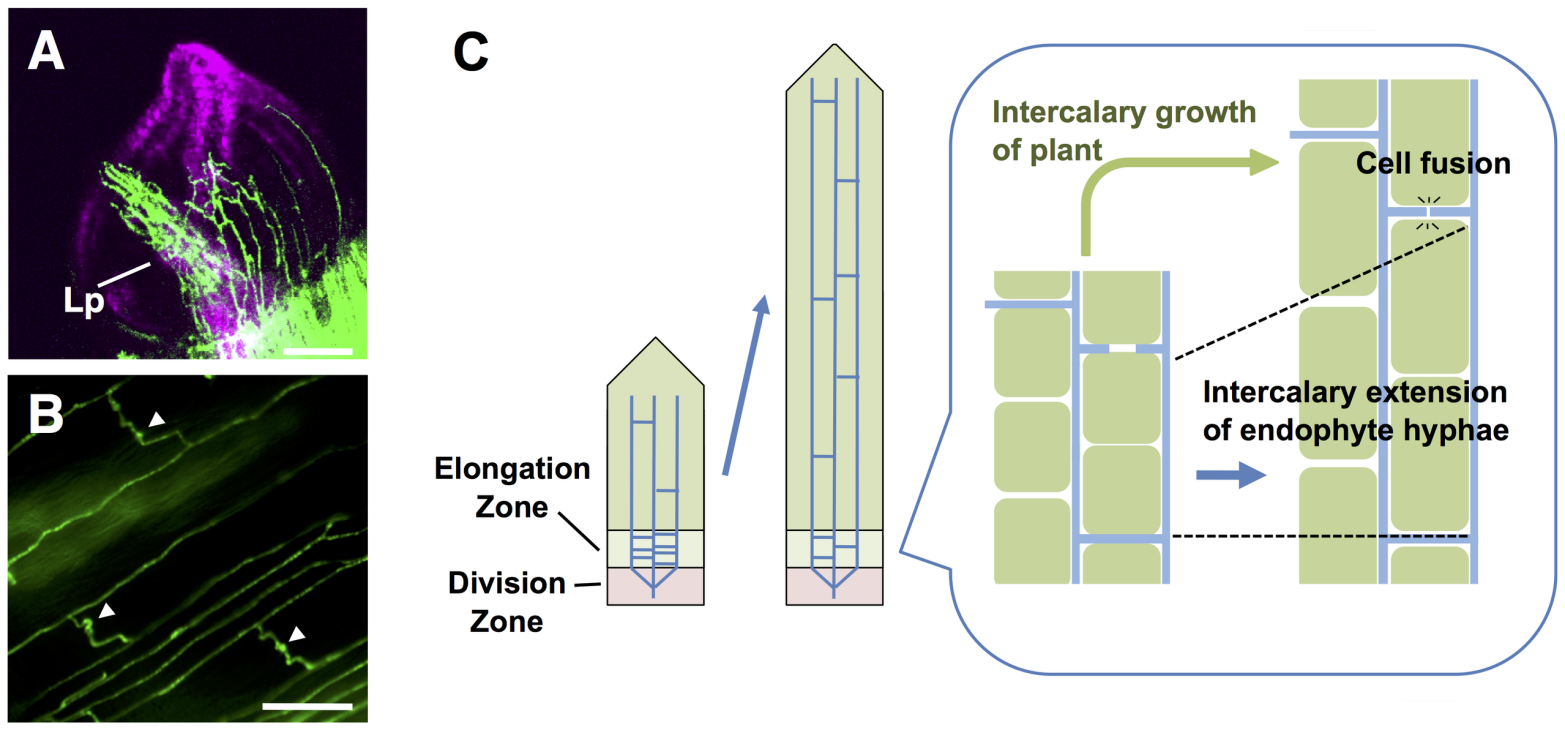
A model showing the growth of endophytic fungus *Epichloë festucae* in host grass plants. **(A)** Hyphal growth of GFP-labeled *E*. *festucae* strain Fl1 in meristematic tissue of perennial ryegrass. LP, leaf primordium. Bar = 100 μm. **(B)** Hyphal growth of GFP-labeled *E*. *festucae* strain Fl1 in pseudostem of perennial ryegrass. Points of hyphal fusion are indicated by arrowheads. Bar = 50 μm. **(C)** Growth zones of a grass leaf and growth pattern of endophyte hyphae. Hyphae of endophyte in host plant is shown as light blue lines. In division zone of grass plant, endophyte hyphae grow by tip growth, whereas middle part of endophyte hyphae extend and divide (intercalary extension) in elongation zone of host plant. Formation of lateral hyphal fusions are often observed (as shown in B). Adapted from Kavanová et al. [66] and Christensen et al. [11].

Another key requirement for *Epichloë* endophytes is strict control of microbial biomass and growth pattern in host plant tissues to maintain symbiotic infection. In contrast to unrestricted extension of infecting hyphae of pathogenic fungi, it has been proposed that *Epichloë* endophytes have a unique mechanism to establish systemic infection in host plants [11, 12] (Fig 1). In meristematic tissue of host plants, the endophytic hyphae extend by tip growth (Fig 1A), but once the tiller of the host is colonized, the hyphae are attached to the plant cells and extend by intercalary growth, which enables the growth of the endophyte to be synchronized with that of the host plant (Fig 1B and 1C). Endophytic hyphae *in planta* are interconnected (Fig 1B), which enables the establishment of a network between parallel-growing hyphae, and control of biomass [13]. Hyphae of some *Epichloë* endophytes are able to grow from the inside of the plant tissue to the outside by means of an expressorium, a newly identified fungal structure that allows penetration of the cuticle, to form a hyphal net on the surface of the leaf [14]. Reactive oxygen species (ROS) produced by NoxA, a specific NADPH oxidase (Nox) isoform, play a crucial role in the symbiotic colonization of the host plant by *E*. *festucae*. Mutants of *noxA* have unrestricted growth inside the host plant, comparable to pathogenic fungi, which results in a severely stunted, and often lethal phenotype of the host plant [15]. These findings indicate that NoxA is required to synchronize the growth of *Epichloë* endophytes *in planta* with the host plant. *noxA* mutants are also defective in hyphal cell-cell fusion, a process that is important for establishing a hyphal network *in planta* [13]. Regulatory components of NoxA, NoxR (human p67^phox^-like protein) and a small GTPase RacA, are also essential for the establishment of symbiotic infection [16, 17]. Homologues of yeast proteins for polarity establishment, BemA and Cdc24, were identified as NoxR-interacting proteins, which suggests that both are potential regulators of ROS production in the fungal Nox complex [18]. *noxR* and *racA* mutants induced stunting of host plants, comparable to the *noxA* mutants [16, 17], while *bemA* mutants induce only a mildly stunted phenotype of the host plant [18]. *noxR* and *racA* mutants are also defective in hyphal cell fusion, whereas *bemA* mutants have a reduction in the frequency of hyphal fusion [13], suggesting that BemA has an accessory role in the Nox complex. Similarly, *E*. *festucae* ProA (a C6 zinc finger transcription factor) and So (a scaffold for a MAP kinase pathway) are involved in hyphal cell fusion, and infection with *proA* and *so* mutants caused stunting of host plants, with a severity similar to that observed in *noxA* mutants [19, 20]. Given that *Neurospora crassa* Nox-1 (NoxA), Nor-1 (NoxR), Rac-1 (RacA), Adv-1 (ProA) and So have been previously reported to be involved in fusion between conidial anastomosis tubes [21–24], these results indicate that hyphal cell fusion is essential for controlling the growth of endophytes in host plants [13].

In this study, we investigated in culture and *in planta* functions of two closely related Rho GTPases, RacA and Cdc42, from the endophytic fungus *E*. *festucae*. Although the functions of Rac and Cdc42 have been characterized in many fungal species, the present study reveals that *E*. *festucae* utilizes these very similar small GTPases in unique ways in the symbiotic interaction with the host plant. RacA and Cdc42 have positive and negative roles in the control of hyphal ROS production. Moreover, critical amino acid residues for specific interactions of small GTPase with BemA and NoxR were identified. Complementation of *racA* and *cdc42* mutants with modified Cdc42 and RacA revealed the significance of specific interactions of Cdc42 and RacA with BemA and NoxR for functional differentiation of these small GTPases in diverse biological processes.

## Results

### Two closely related Rho GTPases, RacA and Cdc42, specifically interact with different components of the fungal Nox complex

Small GTPases are universal molecular switches for eukaryotic cells to regulate a broad range of physiological processes [25–28]. In filamentous fungi, there are 5 subgroups of small GTPases, namely Rab, Arf, Rho and Ras and Ran. Thirty-one genes for small GTPases can be identified from the genome sequence of the endophytic fungus *E*. *festucae* (S1 Fig and S1 Table). Phylogenetic analysis of small GTPases from Ascomycota fungi, including *E*. *festucae*, *N*. *crassa*, *Fusarium graminearum*, and *Magnaporthe oryzae*, revealed that most members of the small GTPases form tight clusters, suggesting conserved functions of small GTPases in Ascomycota fungi (S1 Fig). Rac is a multifunctional small GTPase belonging to the Rho subgroup. One of its conserved functions among mammalian, plant and fungal Rac, is the activation of NADPH oxidases [17, 29, 30]. *E*. *festucae* RacA has been shown to interact with NoxR, an ortholog of the human Nox activator p67^phox^, which also has a key role in regulating ROS production during symbiotic infection [16].

**Fig 2:**
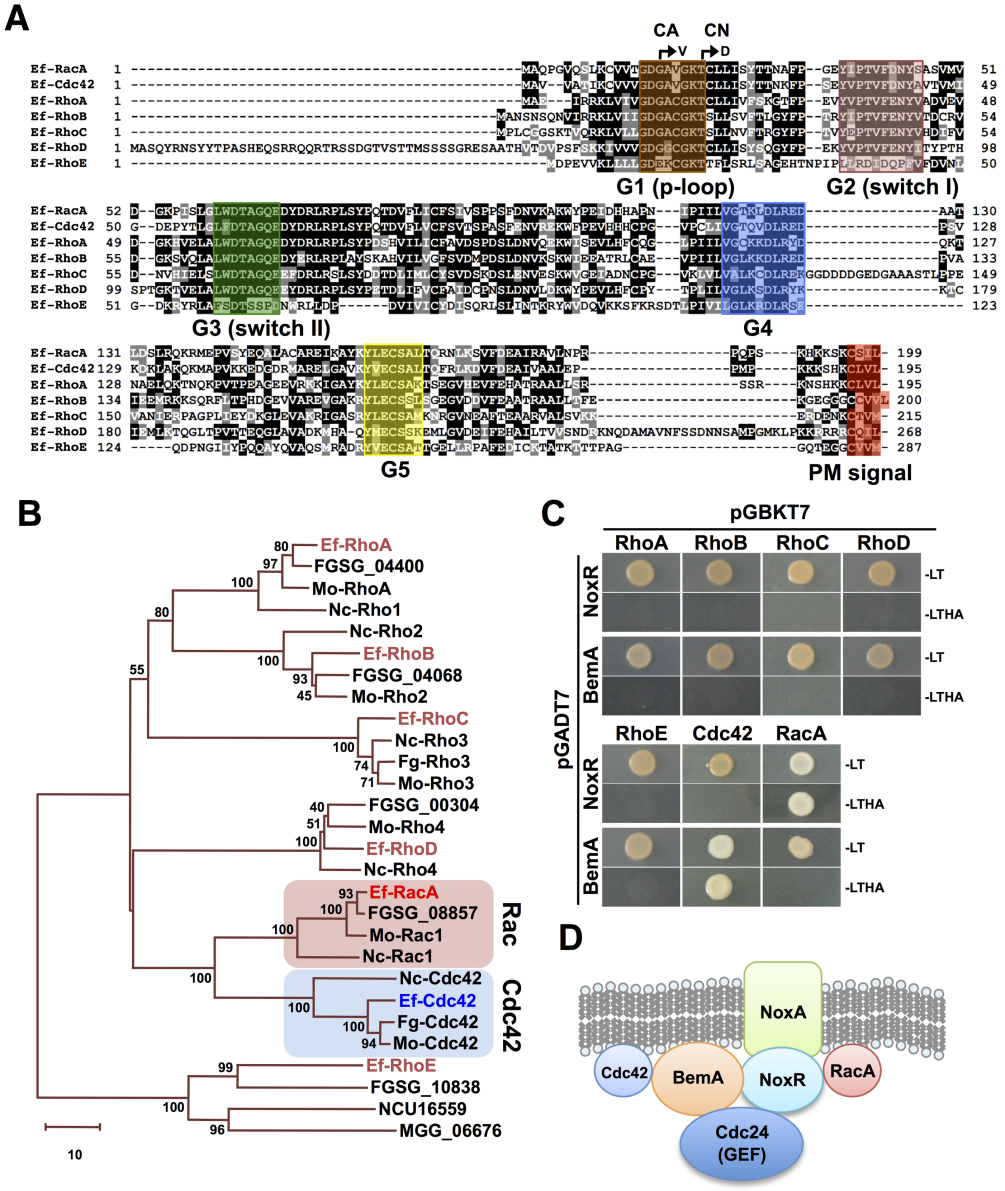
Specific interactions of *Epichloë festucae* Rho GTPases, RacA and Cdc42, with components of Nox complex. **(A)** Alignment of the deduced amino acid sequences for *E*. *festucae* Rho GTPases. Conserved domains among Rho GTPases are boxed. Amino acid substitutions introduced for constitutive active (CA) and negative (CN) form of small GTPases are indicated by arrows. **(B)** Phylogenetic Analysis of Rho GTPase from *E*. *festucae*. The tree was prepared by the neighbor-joining method (Saitou and Nei, 1987). The scale bar corresponds to 10 estimated amino acid substitutions per site. Numbers at the nodes indicate the percentage of 1000 bootstrap replicates that supported each labeled interior branch. Ef; *Epichloë festucae*, Fg; *Fusarium graminearum*, Mo; *Magnaporthe oryzae*, Nc; *Neurospora crassa*. **(C)** Yeast two-hybrid assays of the interactions between *E*. *festucae* NoxR, BemA and Rho GTPases. Rho GTPases have mutation in C-terminal plasma membrane localization signal. Yeast strain AH109 was transformed with prey and bait vector as indicated and plated on to SD medium lacking leucine and tryptophan (-L/-T) or lacking leucine, tryptophan, histidine and adenine (-L/-T/-H/-A). Growth on the latter indicates an interaction between bait and prey. **(D)** A Model for interactions of Cdc42, RacA with components of Nox complex.

Seven genes encoding *E*. *festucae* Rho GTPases were identified (Fig 2, S1 Fig and S1 Table). As Rho GTPases share similar domain structures (Fig 2A), we tested for possible interactions between the 7 Rho GTPases and NoxR by performing yeast two-hybrid assays. As cysteine residues near the C-terminus of Rho GTPases, could prevent the yeast two-hybrid assay (as they are geranylgeranylation sites necessary for plasma membrane localization, Fig 2A) [31], a cysteine to alanine mutation was introduced into each of the Rho GTPases. In this assay, an interaction between NoxR and RacA was observed, but not with the other Rho GTPases (Fig 2C). Interaction assays with BemA, another component of the Nox complex (Fig 2D), and 7 Rho GTPases showed binding between BemA and Cdc42, a Rho GTPase very closely related to RacA (Fig 2C). Further interaction assays for truncated BemA derivatives with Cdc42 showed that Cdc42 interacts with a domain between the second SH3 and the PX domain of BemA (S2A Fig). Further interaction assays of RacA, Cdc42, and Nox components confirmed that NoxR, BemA and Cdc24 can interact with each other as previously reported [18] (S2B Fig), and RacA and Cdc42 specifically interact with NoxR and BemA, respectively (Fig 2D and S2 Fig).

### Bimolecular fluorescence complementation analysis for interactions between BemA, NoxR and small GTPases

**Fig 3:**
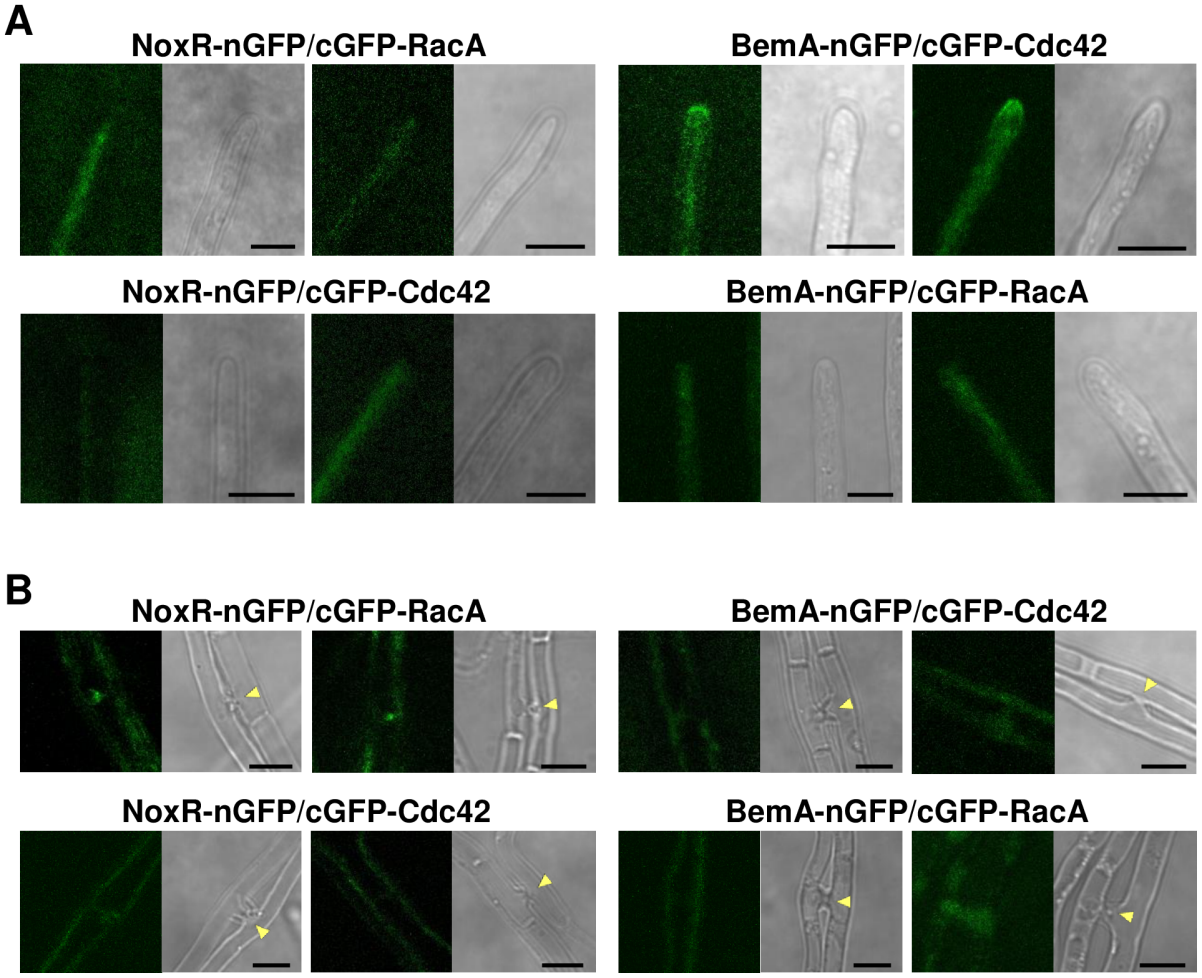
Bimolecular fluorescence complementation analysis of interaction between BemA, NoxR, Cdc42 and RacA in *Epichloë festucae*. BemA, NoxR, Cdc42 and RacA tagged with nGFP (2–174) or cGFP (175–239), were expressed in *E*. *festucae* hyphae under the control of their native promoters as indicated. GFP fluorescence was observed at hyphal tips (A) or sites of hyphal cell fusion (B). Arrowheads indicate hyphal fusions. Bars = 5 μm.

To investigate the interactions of BemA, NoxR, RacA and Cdc42 in hyphae of *E*. *festucae*, we fused the sequences of the N- or C-terminal portions of GFP (nGFP or cGFP) to the genes coding for these proteins, and then assessed their interactions in hyphal cells by bimolecular fluorescence complementation (BiFC) assay [32]. When BemA-nGFP and cGFP-Cdc42 were co-expressed under control of their native promoters, fluorescence was preferentially detected at the hyphal tips, indicating a tip-localized interaction between BemA and Cdc42 (Fig 3A). Interaction at hyphal tips was never observed for the combination of BemA-RacA, NoxR-RacA and NoxR-Cdc42 (Fig 3A). In the case of the combination of NoxR and RacA, localized fluorescence was detected at the initiation sites of hyphal cell fusion, whereas interaction between BemA-Cdc42, NoxR-Cdc42 or BemA-RacA was not detected at the sites of cell fusion (Fig 3B).

### Active form of RacA and Cdc42 Localize at hyphal tips of endophyte

**Fig 4:**
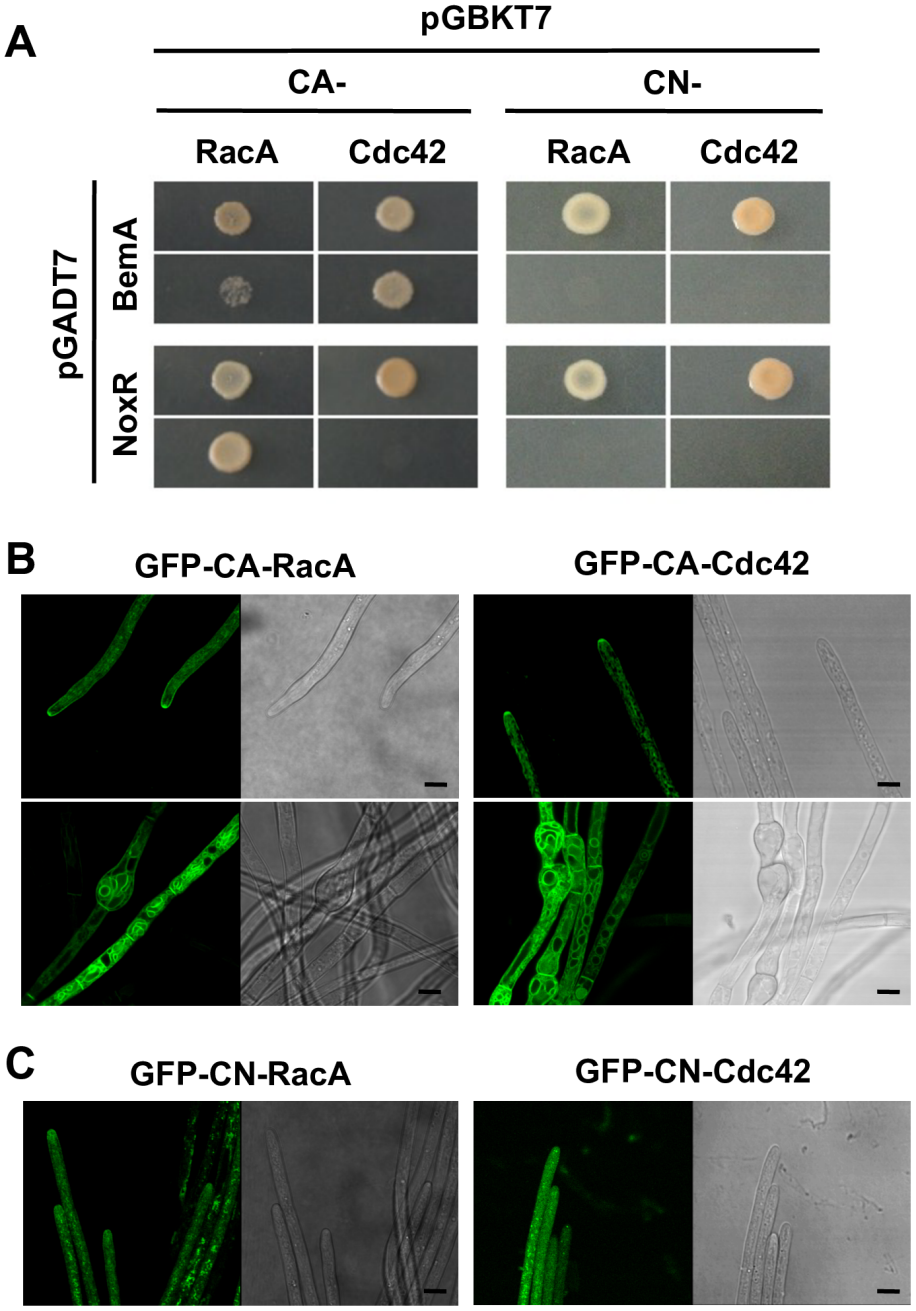
Active form of RacA and Cdc42 can interact with components of fungal Nox complex. **(A)** Yeast two-hybrid assays of the interactions between *Epichloë festucae* constitutive active (CA-) or negative (CN-) form of RacA and Cdc42 with NoxR or BemA. All Rho GTPase derivatives have a mutation (cysteine to alanine) in C-terminal plasma membrane localization signal. Yeast strain AH109 was transformed with prey and bait vector as indicated and plated on to SD medium lacking leucine and tryptophan (-L/-T) or lacking leucine, tryptophan, histidine and adenine (-L/-T/-H/-A). Growth on the latter indicates an interaction between bait and prey. **(B, C)** Subcellular localization of CA- or CN- form of RacA and Cdc42 in *E*. *festucae* hyphae. GFP-tagged RacA or Cdc42 were expressed in *E*. *festucae* under the control of the Tef promoter. Bars = 5 μm.

By introducing a single amino acid substitution, constitutive-active (CA, GTP bound form) and -negative (CN, GDP bound form) forms of Rho GTPase can be produced [17, 33] (Fig 2A). Using CA and CN forms of RacA and Cdc42, interactions with NoxR and BemA were assessed by the yeast two hybrid assay (Fig 4). Interactions between CA-RacA and NoxR, or CA-Cdc42 and BemA were detected, whereas the CN form of RacA and Cdc42 did not bind with NoxR and BemA respectively, indicating that only active Rho GTPases can interact with their effectors (Fig 4A). Relatively weak binding between CA-RacA and BemA was also detected. CA or CN forms of RacA and Cdc42 were labeled with GFP and expressed in *E*. *festucae* under the control of a constitutive Tef promoter [34]. The CA form, but not the CN form, of RacA and Cdc42 localized at hyphal tips (Fig 4B and 4C). The vacuolar membrane was also labeled by GFP-CA-RacA and GFP-CA-Cdc42. Expression of both CA-RacA or CA-Cdc42 caused irregular swelling of hyphae near the hyphal septa, indicating that active RacA and Cdc42 share similar hyphal morphology functions (Fig 4B). Expression of CA-RacA or CA-Cdc42 also caused swelling of hyphae in *noxR* or *bemA* mutant background (S3 Fig), indicating that induction of hyphal swelling by expression of CA-RacA or CA-Cdc42 is independent of NoxR and BemA. Hyphae expressing GFP-CN-RacA and GFP-CN-Cdc42 had a dispersed punctate pattern of fluorescence in hyphal cells, but there was no obvious effect on hyphal growth and morphology by the expression of these forms of the GTPases (Fig 4C).

### Overlapping and contrasting phenotypes of *E*. *festucae racA* and *cdc42* mutants on morphology and polarized growth of hyphae in axenic culture

To investigate the roles of Cdc42 and RacA in *E*. *festucae* mycelial growth, as well as symbiotic infection and ROS production, a *cdc42* replacement construct, pNPP50 was prepared, and recombined into the genome of *E*. *festucae* strain Fl1 (S4A Fig). PCR screening identified three transformants (#48, #80 and #234) that had patterns consistent with targeted replacement events. DNA gel blot analysis of genomic digests of the three transformants confirmed that all transformants contained a replacement at the *cdc42* locus (S4B Fig), but transformants #48 and #234 had additional integrations of the KO construct. Therefore, transformant #80 was used for all subsequent analysis.

**Fig 5:**
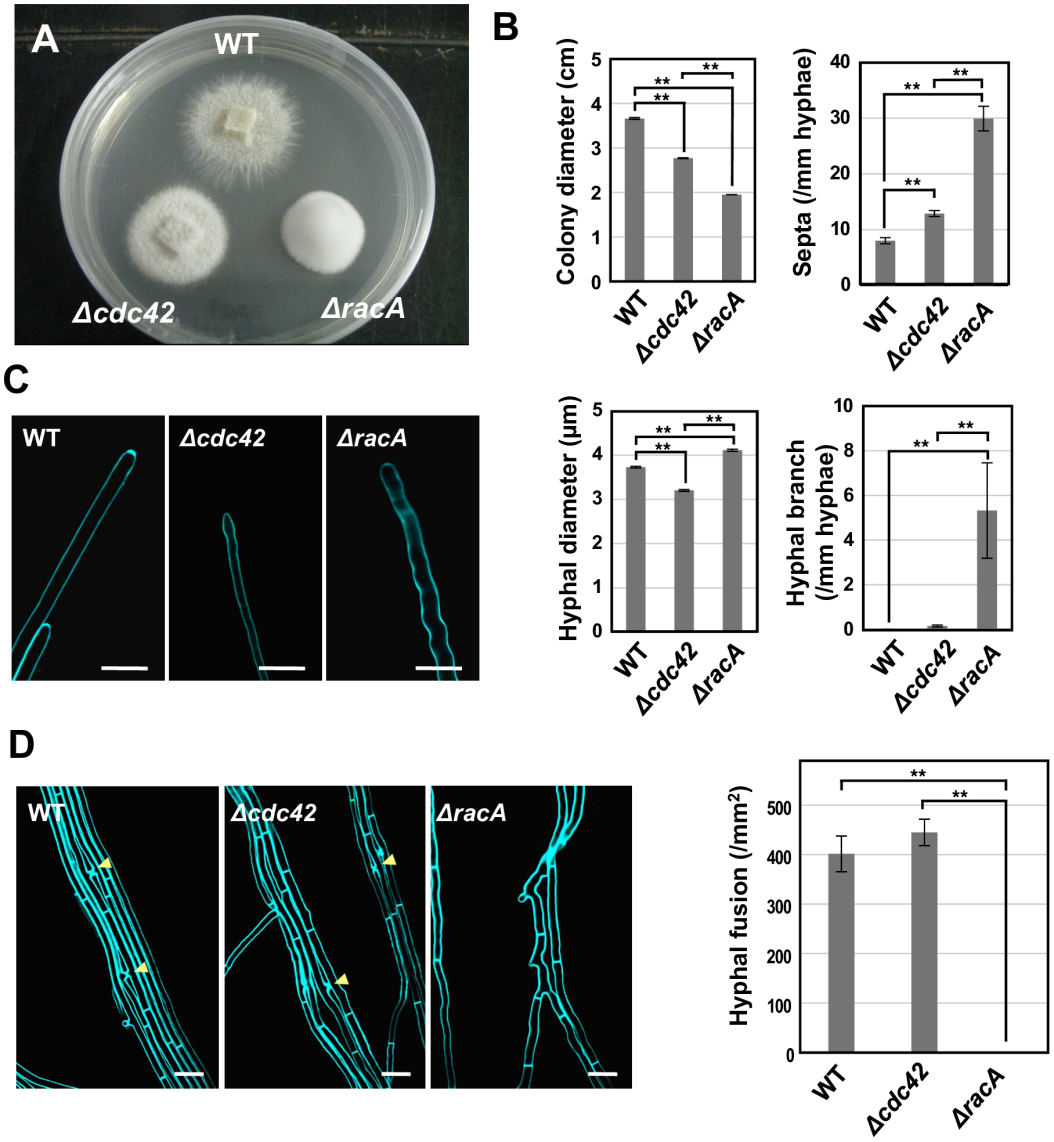
Overlapping and distinct pheynotypes of *Epichloë festucae cdc42* and *racA* mutants in axenic culture. **(A)** Colony morphology of *E*. *festucae* wild type (WT), *cdc42* and *racA* mutants on PDA grown for 12 days. **(B)** Colony diameter, hyphal branch, hyphal diameter and septa formation of *E*. *festucae* WT, *cdc42* and *racA* mutants grown on PDA for 12 days. Data are means ± standard error. n = 10 for colony diameter, n = 20 for hyphal branch, hyphal diameter and septa formation. **(C)** Hyphal tip growth of WT, *cdc42* and *racA* mutant on water ager. Hyphae of endophyte strains were stained with calcofluor white and monitored with confocal laser microscopy. Bars = 10 μm. **(D)** Hyphal fusion of *E*. *festucae* WT, *cdc42* and *racA* mutants grown on water agar. (left) *E*. *festucae* strains were grown on water agar for 12 days, stained with calcofluor white and monitored with confocal laser microscopy. Arrowheads indicate hyphal fusions. Bars = 10 μm. (right) The number of hyphal fusions of *E*. *festucae* strains grown on water agar were counted using a fluorescence microscope after staining with calcofluor white. Data are means ± standard error from 30 sites from three colonies of each strain. Data marked with asterisks are significantly different as assessed by two-tailed Student’s *t* tests: **P < 0.01.

While mutation of *racA* causes a significant increase of aerial hyphae and reduced radial growth in axenic culture (as previously reported [13, 17]), the *cdc42* mutant had only a slight decrease in radial growth and a slight increase in aerial hyphae (Fig 5A and 5B). Microscopic analysis revealed that the *racA* mutant had a significant increase in hyphal septation compared with wild type, while the *cdc42* mutant showed a slight increase of septal formation (Fig 5B). Hyphal volume per compartment is reduced in both *cdc42* and *racA* mutants compared with that of wild type (S5 Fig). When grown on nutrient poor water agar, hyphal tips of both *racA* and *cdc42* mutants showed meandering growth in contrast to the straight hyphae of wild type (Fig 5C), indicating that both RacA and Cdc42 are at least partially involved in the maintenance of hyphal polarity. On nutrient rich PDA agar, irregular hyphal growth was observed for the *racA* mutant but not for the *cdc42* mutant (S6 Fig). These results show that the, *cdc42* mutant has several growth defects similar to *racA* mutant, but all phenotypes were less-severe.

There were some morphological phenotypes specific to either the *racA* or *cdc42* mutant. Hyphae of the *racA* mutant were significantly larger in diameter than wild type. In contrast, the hyphal diameter of the *cdc42* mutant were smaller than wild type (Fig 5B). The amount of hyphal branching was increased in the *racA* mutant, but not significantly affected in the *cdc42* mutant (Fig 5B). Wild type *E*. *festucae* grown on water agar frequently undergoes hyphal cell-cell fusion [13]. As previously reported, hyphae of the *racA* mutant are unable to undergo cell-cell fusion, but in contrast the *cdc42* mutant formed hyphal fusions at a frequency similar to wild type [13] (Fig 3D). Since the regulation of the cytoskeleton is a common function of Rho-type small GTPases [35], the distribution of actin was monitored in wild type and mutant strains by expression of LifeAct-GFP [36] (S7 Fig). In wild type, accumulation of actin patches, was detected near the wall of the hyphal cell approx. 3 μm behind the tip apex (S7 Fig). In the *cdc42* mutant, actin patches were mainly localized at the tip apex of the hyphae, whereas the sites of actin accumulation were closer to the tip apex of hyphae in the *racA* mutant compared with wild type (S7 Fig).

### RacA and Cdc42 have opposite roles for ROS production

**Fig 6:**
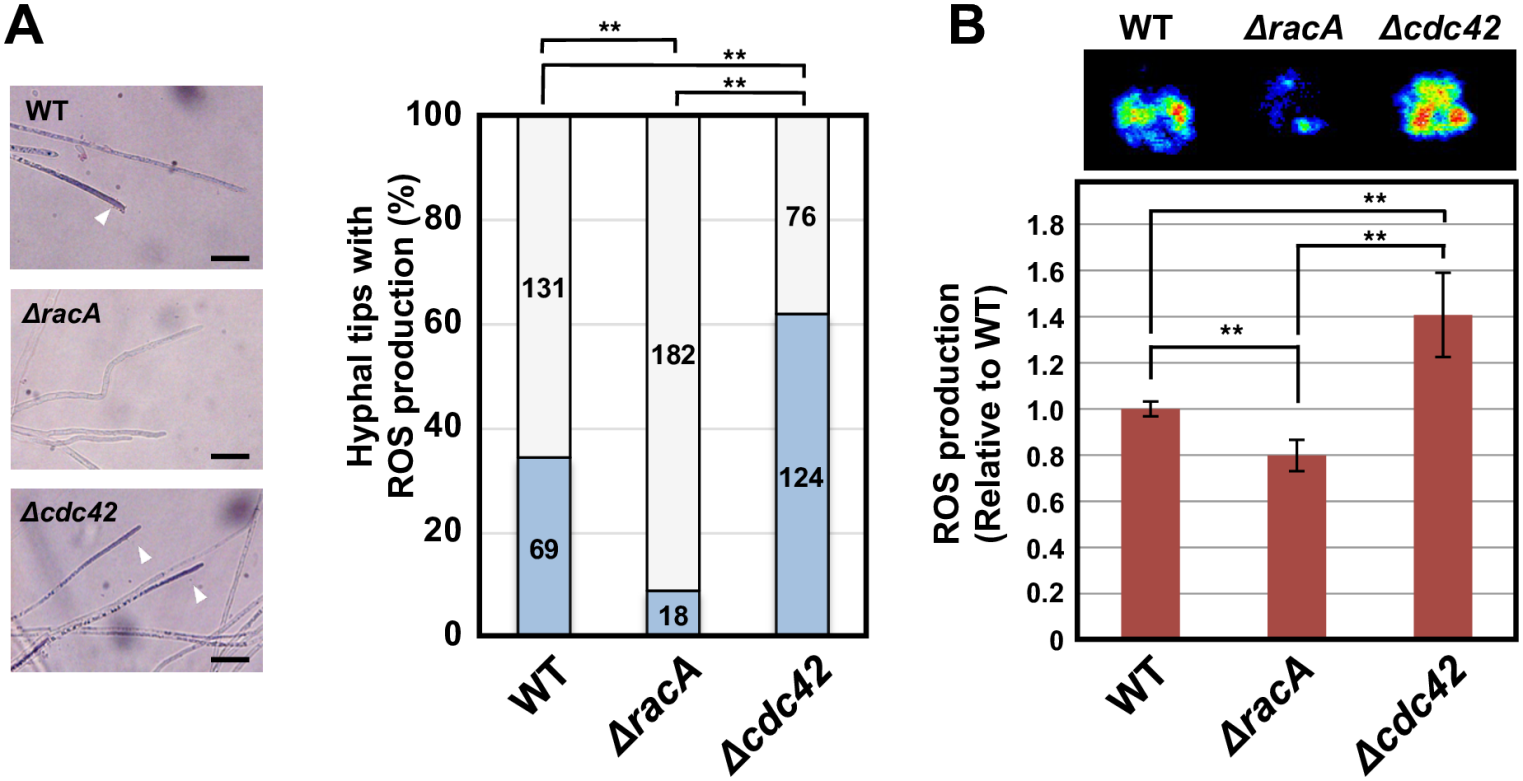
Reactive oxygen species production of *Epichloë festucae* wild type, *racA* and *cdc42* mutants. **(A)** (left) Light micrographs showing production of reactive oxygen species (ROS), as detected by nitroblue tetrazolium (NBT) staining, in *E*. *festucae* WT, *racA* (*ΔracA*) and *cdc42* mutant (*Δcdc42*) mutant. Hyphal tips stained with NBT were shown by arrow heads. Bar = 20 μm. (right) Frequency of NBT-stained hyphal tips of wild-type, *racA* mutant and *cdc42* mutant. Numbers in column indicate hyphal tips counted. *E*. *festucae* strains were grown on PDA for 7 days and stained with NBT for 6 hours. Data marked with asterisks are significantly different as assessed by one-tailed Mann-Whitney U tests: **P < 0.01. **(B)** L-012-mediated detection of ROS production by *E*. *festucae* WT, *racA* mutant and *cdc42* mutant. Colony edge of endophyte strains grown on PDA was treated with L-012 and ROS production was detected as chemiluminescence (top). Value of chemiluminescence relative to wild type was scored. Data are means ± standard error. n = 179. Data marked with asterisks are significantly different as assessed by two-tailed Student’s *t* tests: **P < 0.01.

The effect of *racA* and *cdc42* deletion on hyphal ROS production was examined (Fig 6). Previously, localized production of ROS at hyphal tips of the endophyte was detected by NBT staining in axenic culture [16]. By counting NBT-stained hyphal tips, production of ROS at hyphal tips was compared between wild type and mutant strains. While the ratio of NBT-stained hyphal tips was approx. 35% for wild type, the percentage of NBT-stained hyphal tips in the *racA* mutant was just 9% (Fig 6A), a result consistent with the reduced production of ROS in the *racA* mutant [17]. In contrast, over 60% of hyphal tips in the *cdc42* mutant were stained by NBT, indicating that deletion of *cdc42* enhances ROS production. To confirm the effects of *racA* and *cdc42* KO on ROS production, we performed luminol-mediated assays to detect ROS production. Production of ROS in culture was measured by chemiluminescence, using the luminol derivative L-012 [37]. We first tested the production of ROS in different parts of the endophyte colony. Highest ROS production was detected at the edge of the colonies (S8 Fig), confirming that ROS production is most active at growing hyphal tips, as detected by NBT-staining. In the *racA* mutant, ROS production was reduced by approx. 20% compared with wild type, whereas ROS production in the *cdc42* mutant was increased by 40% (Fig 6B).

### *E*. *festucae* Cdc42 is required for *in planta* hyphal growth of the endophyte synchronized with the host plant

**Fig 7:**
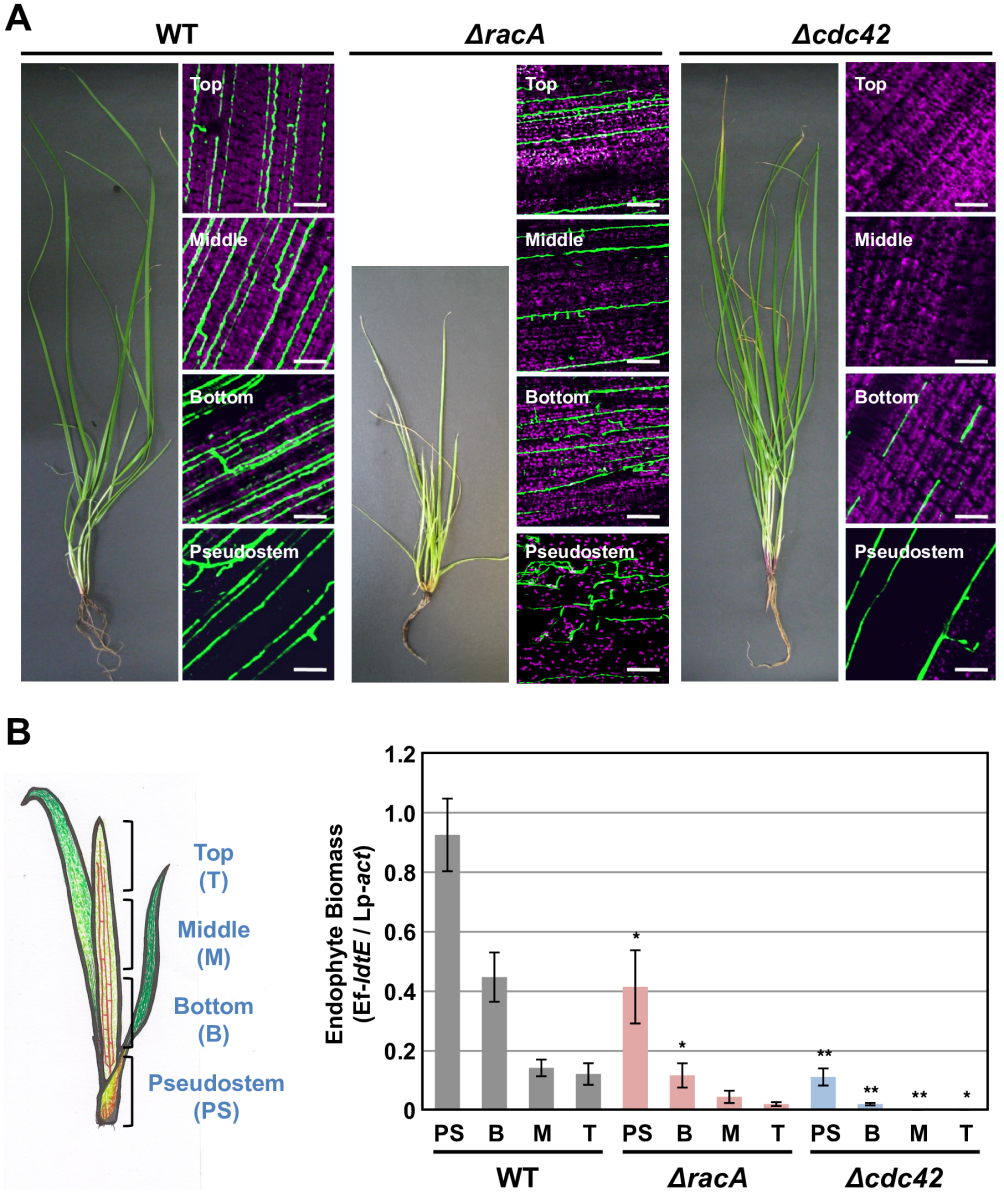
Cdc42 is essential for systemic infection of *Epichloë festucae* in perennial ryegrass. **(A)** Perennial ryegrass was inoculated with *E*. *festucae* wild type (WT), *racA* mutant (*ΔracA*) or *cdc42* mutant (*Δcdc42*) expressing GFP. Infection of endophyte strain in top, middle and bottom part of leaf blade and pseudostem of host plant was monitored by confocal microscopy. Bars = 50 μm. **(B)** Biomasses of *E*. *festucae* in perennial ryegrass leaves were determined by quantitative PCR 2 month after inoculation as relative amount of the endophyte Ef-*ldtE* gene to that of perennial ryegrass Lp-*act* gene. Data are means ± standard error (n = 6 for WT and n = 3 for *cdc42* and *racA* mutants). Data marked with asterisks are significantly different from control (biomass of wild type in corresponding plant tissues) as assessed by the two-tailed Student’s *t* test: *P < 0.05 and ** 0.01.

**Fig 8:**
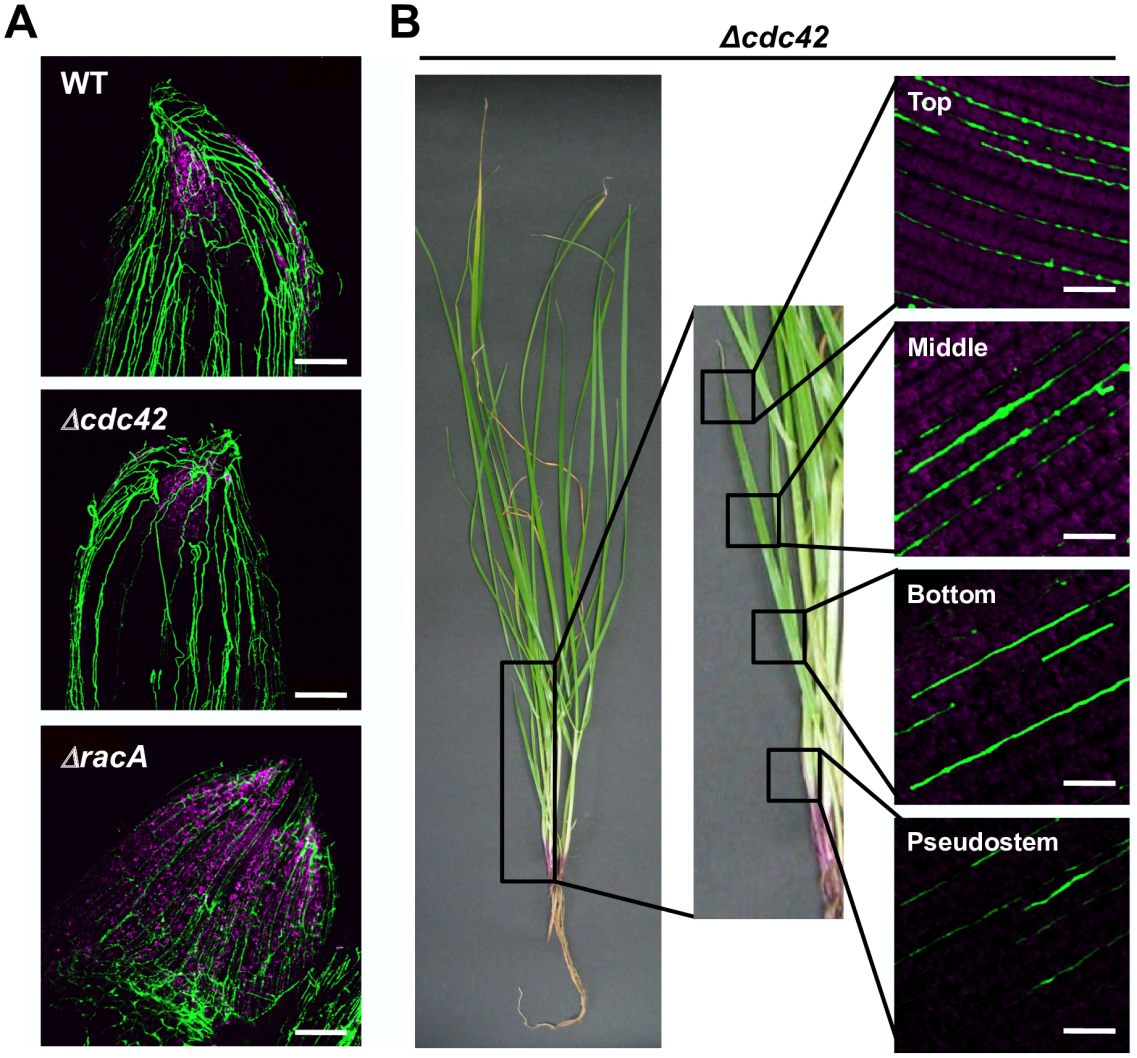
*cdc42* mutant can systemically colonize young tissues of perennial ryegrass. **(A)** Lateral bud of perennial ryegrass infected with *Epichloë festucae* wild type (WT), *cdc42* mutant (*Δcdc42*) and *racA* mutant (*ΔracA*) expressing GFP. Bars = 100 μm. **(B)** *cdc42* mutant can systemically infect young tillers of perennial ryegrass. Bars = 50 μm.

To examine the effects of *racA* and *cdc42* KO on the colonization of the host plant, constitutively expressed green fluorescent protein (GFP) was introduced into the wild type, *racA* and *cdc42* mutant strains. Perennial ryegrass infected with the *racA* mutant showed the typical stunted phenotype as previously observed with the *racA* mutant [17], whereas inoculation with wild type and *cdc42* mutant caused no obvious growth phenotype in the host plant (S9 Fig). Hyphal growth in these associations was examined by confocal laser scanning microscopy (Fig 7). Hyphae of the wild type strain expressing GFP were mainly orientated parallel to the longitudinal axis of leaf tissues, with occasional hyphal branching (Fig 7A). Hyphal growth of the *racA* mutant *in planta* showed a relatively unregulated pattern with hyphae distributed throughout the leaf from the pseudostem to the upper regions of the leaf blade, as found for wild type, but the pattern of growth was significantly altered. In contrast to wild type, hyphae of the *racA* mutant were frequently branched, were convoluted and unable to fuse (Fig 7A). In contrast, hyphae of the *cdc42* mutant were observed only in the pseudostem and the lower part of the leaf blade (Fig 7A). Although the growth pattern of the *cdc42* mutant in the pseudostem was similar to that of the wild type strain, the biomass of the *cdc42* mutant hyphae was relatively low. In the lower part of the leaf blade, hyphae of *cdc42* mutant were fragmented (Fig 7A). In the meristematic tissue of host plants, the degree of hyphal colonization for wild type, *cdc42* mutant and *racA* mutant was comparable (Fig 8A). In younger tillers of the host plant, hyphae of the *cdc42* mutant were detected in the upper, central and lower parts of the leaf blades as well as in the pseudostems, though occasional fragmentation of hyphae was observed (Fig 8B). Given that vegetative hyphae of *E*. *festucae* are proposed to extend by intercalary growth in the host leaf tissues (Fig 1) [11], fragmentation of the *cdc42* mutant hyphae in older leaves may indicate that Cdc42 is required for endophyte-specific intercalary hyphal growth during the extension of host leaves. Quantitative PCR analysis confirmed that the biomass of the *cdc42* mutant is significantly reduced in pseudostem and the lower part of leaf blades compared with that of wild-type, with no infection detected in the central and upper parts of the host plant (Fig 7B), as was expected from the observed colonization phenotype. Unexpectedly, the overall biomass of the *racA* mutant was reduced compared to wild type (Fig 7B), possibly because of an uneven distribution of the *racA* mutant in host tissues (Fig 7A). Together, these results show that Cdc42 is required for systemic infection of *E*. *festucae* within the growing host plant.

### Identification of amino acids essential for specific interactions between Cdc42 and BemA or RacA and NoxR

**Fig 9:**
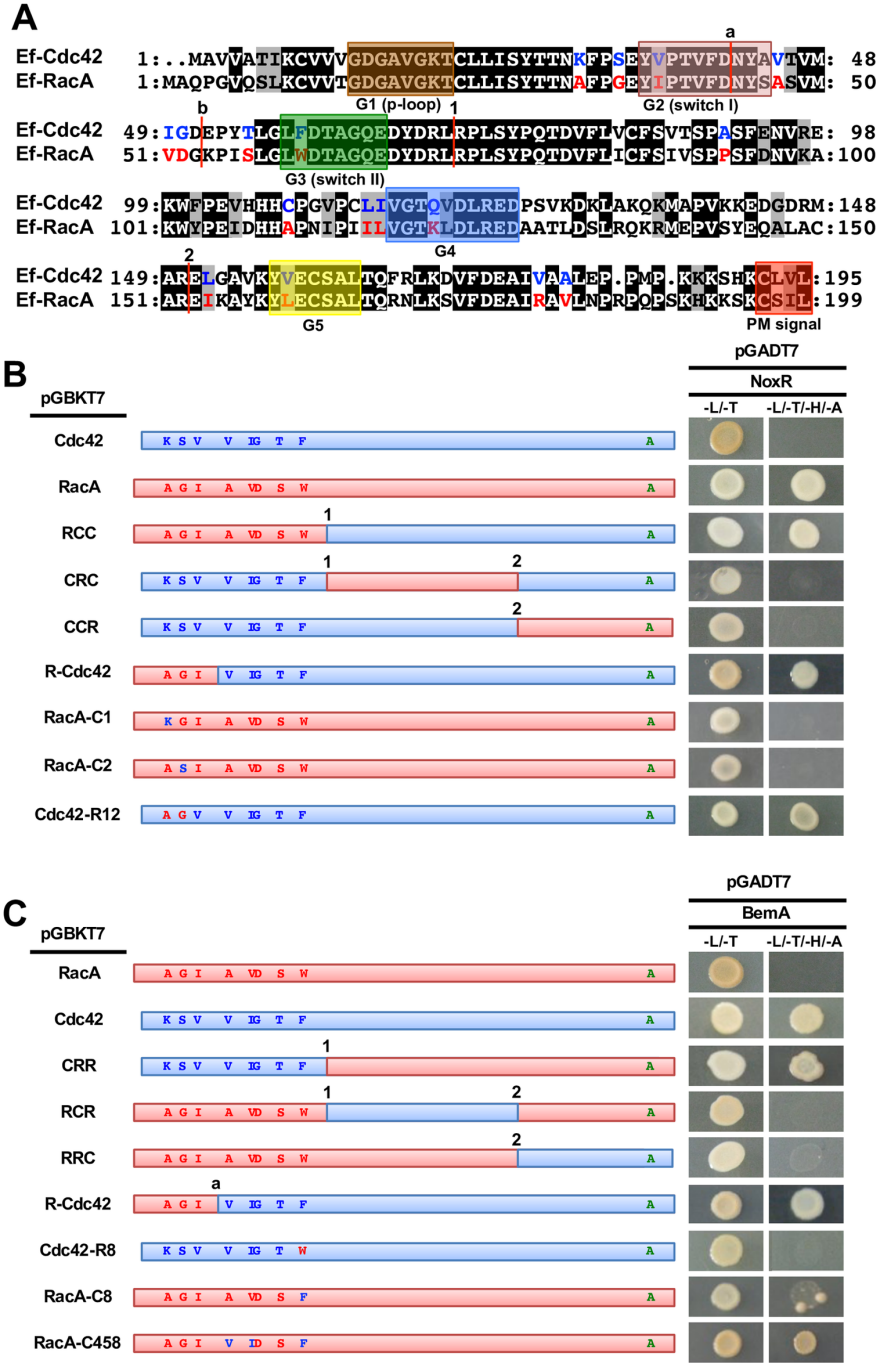
Identification of essential amino acids for specific interactions between Cdc42-BemA or RacA-NoxR. **(A)** Alignment of the deduced amino acid sequences for *Epichloë festucae* Cdc42 and RacA. Conserved domains among Rho GTPases are boxed. Amino acids specifically conserved among fungal Cdc42 and RacA are indicated by blue and red letters, respectively. **(B)** Yeast two-hybrid assays of the interactions between *E*. *festucae* NoxR and chimeric or mutated Cdc42 and RacA. **(C)** Yeast two-hybrid assays of the interactions between *E*. *festucae* BemA and chimeric or mutated Cdc42 and RacA. Rho GTPases have mutation in C-terminal plasma membrane localization signal. Yeast strain AH109 was transformed with prey and baid vector as indicated and plated on to SD medium lacking leucine and tryptophan (-L/-T) or lacking leucine, tryptophan, histidine and adenine (-L/-T/-H/-A). Growth on the latter indicates an interaction between bait and prey.

To investigate the mechanisms that determine the distinct roles of RacA and Cdc42, we set out to identify amino acid residues essential for the binding of these small GTPases to Nox components. The 3D structures of RacA and Cdc42 are predicted to be very similar (S10 Fig), as they contain the same set of conserved domains for functions of small GTPases. These conserved domains include the GTP-binding and hydrolysis domains (G1-G5), as well as a C-terminal CaaX motif (a, any aliphatic amino acid), which is predicted to allow association with the plasma membrane after geranylgeranylation of the cysteine residue (Fig 9) [38, 39]. A multiple sequence alignment of Cdc42 and RacA from 15 fungal species showed that there were 17 conserved amino acids in each of the proteins that distinguished Cdc42 from RacA (S11 Fig and Fig 9A). To identify amino acids essential for specific interactions between Cdc42 and BemA or RacA and NoxR, yeast two-hybrid assays were carried out using various chimeric Cdc42 and RacA proteins. Cdc42 and RacA were divided into 3 portions (Fig 9A, demarcated by 1 and 2), and chimeric genes with different combinations thereof were cloned into the bait vector pGBKT7. These chimeric genes were named, following a simple system which reflects the order of the constituent portions. For example, the name CRC defines the chimeric gene with an N-terminal fragment from Cdc42, a central portion from RacA and a C-terminal portion from Cdc42 (Fig 9B). Interactions of NoxR with RCC, but not with CRC and CCR, were detected, indicating that the N-terminal portion of RacA is required for interaction with NoxR (Fig 9B). Similarly, BemA interacted with CRR, but not with RCR or RRC, indicating that the N-terminal portion of Cdc42 is required for interaction with BemA (Fig 9C). Given that 8 of the 17 conserved amino acid residues that distinguish RacA from Cdc42 were found in the N-terminal region (Fig 9A and S11 Fig), RacA or Cdc42 derivatives with substitutions in these 8 amino acids were generated for further analysis. A mutated RacA with the first conserved amino acid substituted with the corresponding amino acid of Cdc42 was named RacA-C1 (Fig 9B). Interaction assays of 8 mutated RacA derivatives with NoxR revealed that RacA-C1 (RacA A32K) and -C2 (RacA G35S), but not RacA-C3, -C4, -C5, -C6, -C7 and -C8, lost binding with NoxR (S12A Fig and Fig 9B). Moreover, Cdc42-R12 (Cdc42 K30A, S33G) can interact with NoxR, which indicates that alanine 32 and glycine 35 in RacA are necessary and sufficient for interaction of RacA/Cdc42 with NoxR (Fig 9B). Interaction assays of 8 mutated Cdc42 derivatives with BemA revealed that only Cdc42-R8 (Cdc42 F59W), but no other Cdc42 point mutations, lost the ability to bind to BemA (S10B Fig and Fig 9C), which indicates that the phenylalanine 59 is essential for interaction of Cdc42 with BemA. Interaction of RacA-C8 (W61F) with BemA did occur, although weaker than the interaction between wild type Cdc42 and BemA (Fig 9C). A series of interaction assays with various mutated RacA revealed that the valine 45 and isoleucine 49 in Cdc42 are also involved in the interaction between Cdc42 and BemA (Fig 9C and S12B Fig). Cdc42 and RacA derivatives were used in the following complementation analyses to investigate the importance of Cdc42-BemA and RacA-NoxR interactions in hyphal growth, ROS production and symbiotic infection by *E*. *festucae* in the host plant.

### Introduction of NoxR-bound Cdc42 cannot recover the morphological defects and ROS production of *racA* mutant in culture

**Fig 10:**
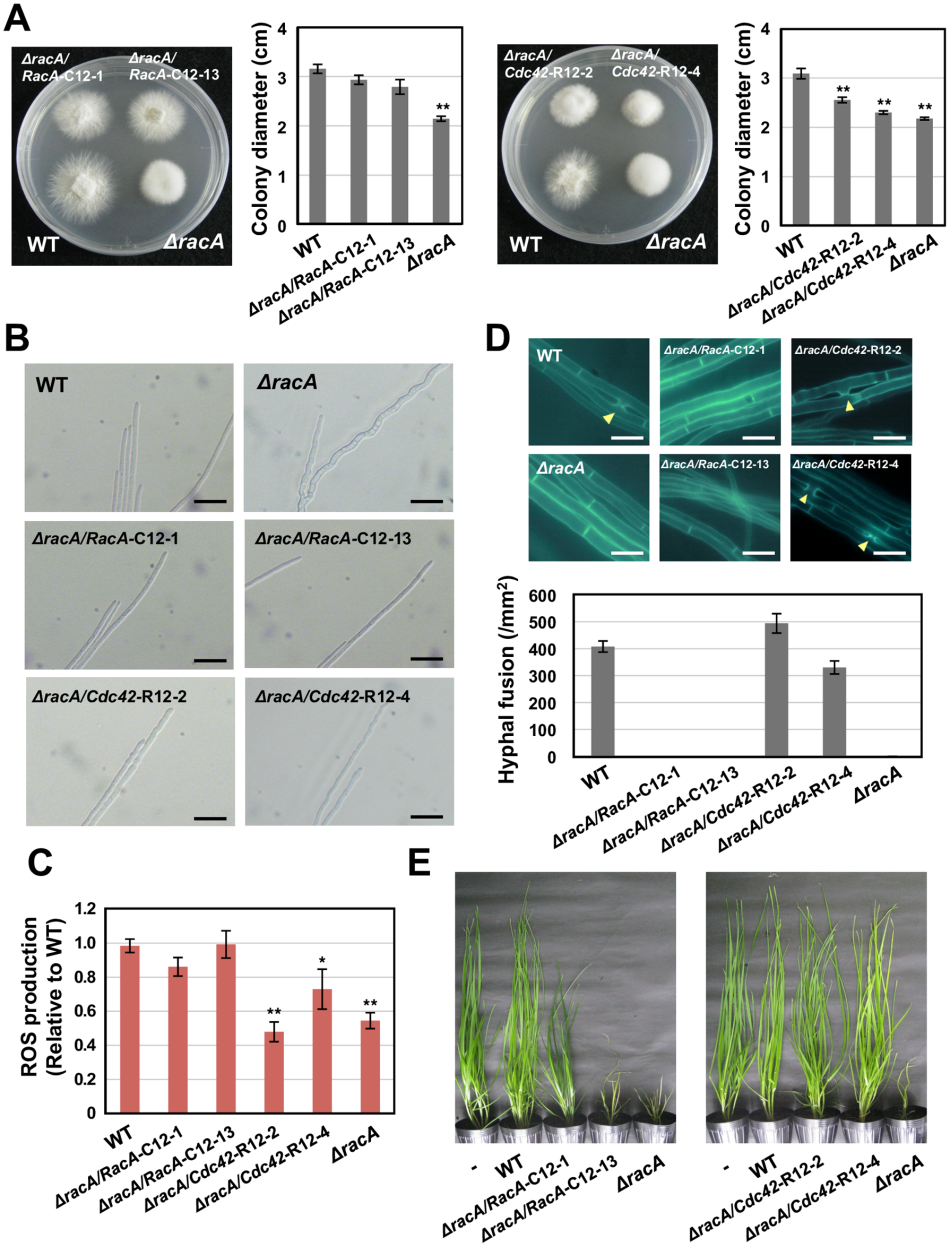
Binding of RacA/Cdc42 to NoxR is required for hyphal fusion and symbiotic infection of *Epichloë festucae*. **(A)** Colony morphology and diameter of *E*. *festucae* wild type (WT), *racA* mutant and complemented strains on PDA grown for 10 days. Data are means ± standard error. n = 9. Data marked with asterisks are significantly different from wild type as assessed by two-tailed Student’s *t* tests: **P < 0.01. **(B)** Hyphal tip growth of WT, *racA* mutant and complemented strains on PD ager for 20 days. Bars = 20 μm. **(C)** L-012-mediated detection of ROS production by *E*. *festucae* WT, *racA* mutant and complemented strains. Colony edge of endophyte strains grown on PDA was treated with L-012 and ROS production was detected as chemiluminescence. Value of chemiluminescence relative to wild type was scored. Data are means ± standard error. n = 18. Data marked with asterisks are significantly different from wild type as assessed by two-tailed Student’s *t* tests: **P < 0.01, *P < 0.05. **(D)** Hyphal fusion of *E*. *festucae* WT, *racA* mutant and complemented strains grown on water (top) *E*. *festucae* strains were grown on water agar for 10 days, stained with calcofluor white and monitored with fluorescence microscopy. Arrowheads indicate hyphal fusions. Bars = 10 μm. (bottom) The number of hyphal fusions of *E*. *festucae* strains grown on water agar. Data are means ± standard error from 30 sites from three colonies of each strain. **(E)** Phenotype of perennial ryegrass infected with *E*. *festucae* WT, *racA* mutant or complemented strains. Photographs were taken approx. 11 weeks after inoculation.

To test the biological significance of NoxR binding for functional differentiation of RacA and Cdc42, either RacA-C12 (no binding with NoxR) or Cdc42-R12 (NoxR binding form) under the control of a constitutive Tef promoter were introduced into the *racA* mutant (Fig 10). It was confirmed that the *racA* mutant, expressing the wild type *RacA* gene under control of the Tef promoter, exhibits wild type phenotypes (S13 Fig). Colonies of *racA* transformed with RacA-C12, were mostly restored to the wild-type morphology, whereas *racA* transformed with Cdc42-R12 retained the phenotype of the *racA* mutant (Fig 10A). Hyphae of the *racA* mutant have a convoluted pattern of growth (Figs 5C and 10B), but introduction of RacA-C12 rescued the wild type phenotype. In contrast, the *racA* mutant transformed with Cdc42-R12 retained the convoluted hyphal phenotype (Fig 10B). These results suggest that RacA binding with NoxR is not required to establish normal polarized hyphal growth of *E*. *festucae* in culture.

Previously we showed that RacA but not NoxR is required for ROS production in axenic culture [16, 17]. To test whether NoxR binding is important for the role of RacA in ROS production of *E*. *festucae* in culture, we measured ROS production from the colony using a luminol based chemiluminescent assay. While *racA* mutants had reduced ROS production, introduction of RacA-C12 was able to restore wild type levels of ROS production (Fig 10C). On the other hand, Cdc42-R12 was unable to rescue ROS production of the *racA* mutant (Fig 10C), suggesting that NoxR binding is not necessary for RacA to induce ROS production from *E*. *festucae* in culture.

### Binding of RacA/Cdc42 to NoxR is essential for hyphal cell fusion and symbiotic infection by the endophyte in host plant

Previously we showed that hyphal cell-cell fusion is crucial for establishing symbiotic infection in the host plant [13]. Although the defects in colony morphology, polarized hyphal growth and ROS production of the *racA* mutant were not rescued by introduction of NoxR-bound Cdc42-R12 (Fig 10A–10C), hyphal fusion of *racA* mutants was restored by complementation with this construct. In contrast, cell-cell fusion was never observed in the *racA* mutant expressing RacA-C12, which cannot bind with NoxR (Fig 10D). To check whether these strains have the ability to establish symbiotic infection, perennial ryegrass seedlings were inoculated with the *racA* mutant expressing either RacA-C12 or Cdc42-R12. Plants infected with *racA*/RacA-C12 had the same stunted phenotype as the *racA* mutant (Fig 10E), indicating that binding to NoxR is essential for RacA to function during symbiotic infection. In contrast, host plants infected with *racA*/Cdc42-R12 grew the same as wild type-infected plants (Fig 10E).

### Mutated RacA with BemA-binding activity partially restores the defects of *cdc42* mutants in systemic infection

**Fig 11:**
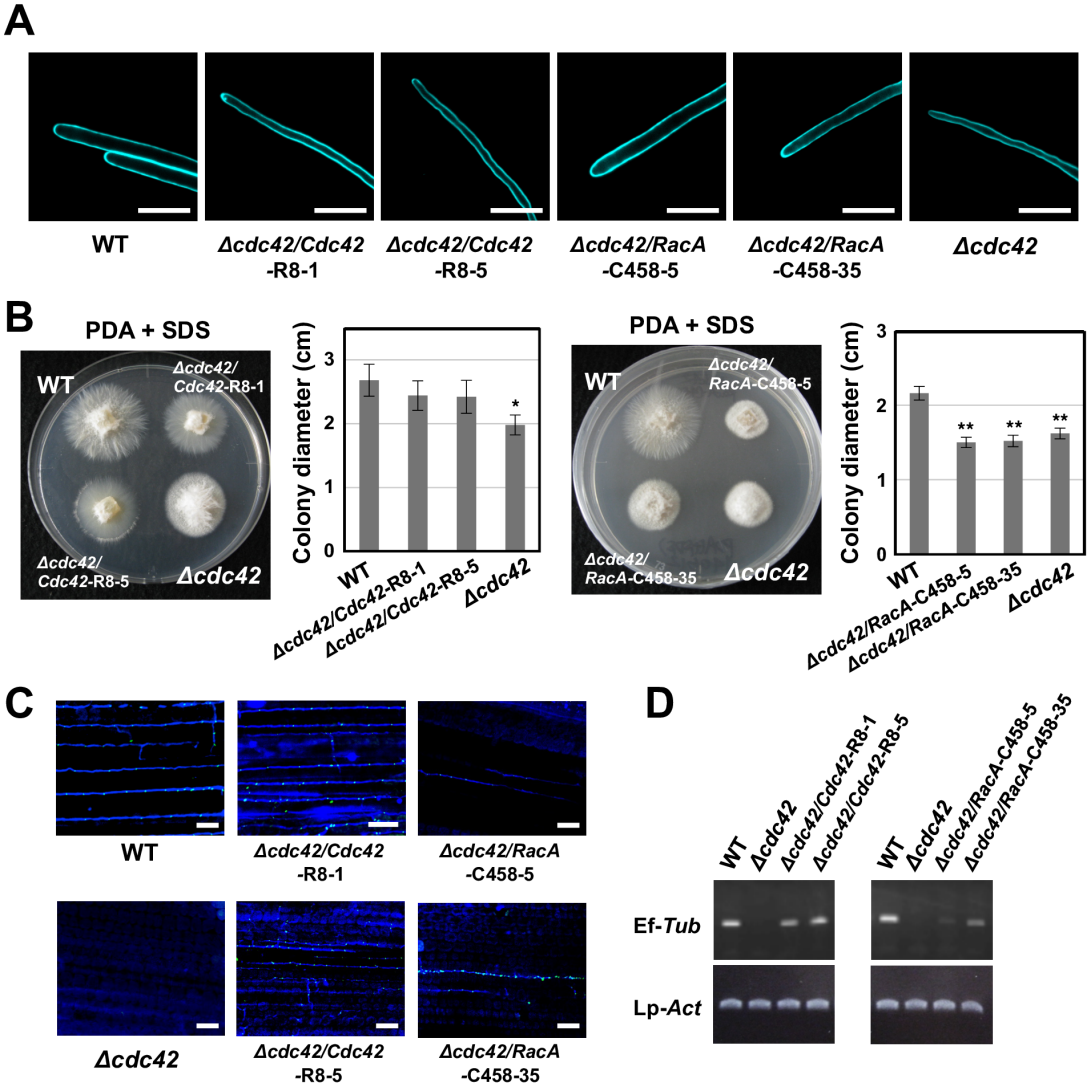
Binding of RacA/Cdc42 to BemA is required for hyphal polarized growth, but not for host infection of endophyte. **(A)** Hyphal tip growth of *Epichloë festucae* wild type (WT), *cdc42* and complemented strains on water ager. Hyphae of endophyte strains were stained with calcofluor white and monitored with confocal laser microscopy. Bars = 10 μm. **(B)** Colony morphology (left) and diameter (right) of *E*. *festucae* WT, *cdc42* mutant and complemented strains on PDA grown supplemented 0.01% SDS for 12 days. Data are means ± standard error. n = 5. Data marked with asterisks are significantly different from wild type as assessed by two-tailed Student’s *t* tests: **P < 0.01, *P < 0.05. **(C)** Colonization of *E*. *festucae* WT, *cdc42* mutant and complemented strains in top part of perennial ryegrass tillers approx. 2 months after inoculation. Hyphae (blue lines) and septa (green dots) were visualized by WG-AF488/aniline blue staining monitored by confocal microscopy. Bars = 40 μm. **(D)** Semi-quantitative PCR detection of endophyte biomass in top part of perennial ryegrass tillers. Specific primes for *E*. *festucae b-tubulin* (Ef-*Tub*) and perennial ryegrass *Actin* gene (Lp-*Act*) were used for detection of endophyte biomass and internal standard, respectively.

To investigate the biological significance of BemA binding for the functional differentiation of RacA and Cdc42, either mutated Cdc42 lacking BemA binding activity (Cdc42-R8) or mutated RacA with BemA-binding activity (RacA-C458), were introduced into the *cdc42* mutant (Fig 11). The hyphae of the *cdc42* mutant had a convoluted pattern of growth in contrast to straight hyphae for the wild type on water agar (Fig 5C), indicating that Cdc42 is required for maintenance of hyphal polarity of *E*. *festucae* under nutrient poor conditions. *cdc42*/Cdc42-R8 had the same defect in hyphal growth as the *cdc42* mutant, whereas hyphae of *cdc42*/RacA-C458 grew straight like wild type (Fig 11A). These results identify the key amino acid residues in Cdc42 that are distinct from RacA that are necessary and sufficient for BemA binding and the polarized hyphal growth function of Cdc42.

The radial growth of the *cdc42* mutant colonies was approx. 80% that of the wild type (Fig 5B). On PDA medium containing 0.01% SDS, the colony growth of *cdc42* mutants was approx. 70% that of wild type (Fig 11B), indicating that *cdc42* mutants are slightly more sensitive to SDS than wild type. Thus, PDA containing 0.01% SDS was used to compare the colony growth of the *cdc42* mutant transformed with various chimeric alleles and restoration of cell wall integrity. Expression of Cdc42-R8 partially restored the colony growth defect of the *cdc42* mutant, whereas colony growth of *cdc42*/RacA-C458 was comparable to the *cdc42* mutant (Fig 11B), which indicates that binding of Cdc42 to BemA is not important for the cell wall integrity function of Cdc42. Perennial ryegrass was inoculated with *cdc42*/Cdc42-R8 or *cdc42*/RacA-C458 to examine the importance of BemA-binding for the function of Cdc42 in symbiotic infection. WG-AF488/aniline blue staining was used to visualize hyphae (in blue) and septa (in green) of the endophyte *in planta* [18]. While wild type endophytes can systemically infect aerial tissue of the host plant, hyphae of the *cdc42* mutant were never detected in the central or upper portions of the leaf blade (Figs 7 and 11C). Host plants inoculated with *cdc42*/Cdc42-R8 were like wild type, with hyphae detected in the upper part of the leaf blade, as in wild type (Fig 11C). It therefore follows, that binding with BemA is not essential for the function of Cdc42 in systemic infection of *E*. *festucae* in the grass host. Moreover, systemic infection with *cdc42*/RacA-C458 was also observed in host plants, though the degree of infection tended to be lower when compared with wild type and *cdc42*/Cdc42-R8 (Fig 11C and 11D). Introduction of wild type RacA into the *cdc42* mutant also partially restored the ability of *E*. *festucae* to infect systemically (S14 Fig). These results indicate that both RacA and Cdc42 contribute to intercalary hyphal growth during the establishment of systemic infection in the host plant, albeit Cdc42 appears to play a more important role than RacA.

## Discussion

**Fig 12:**
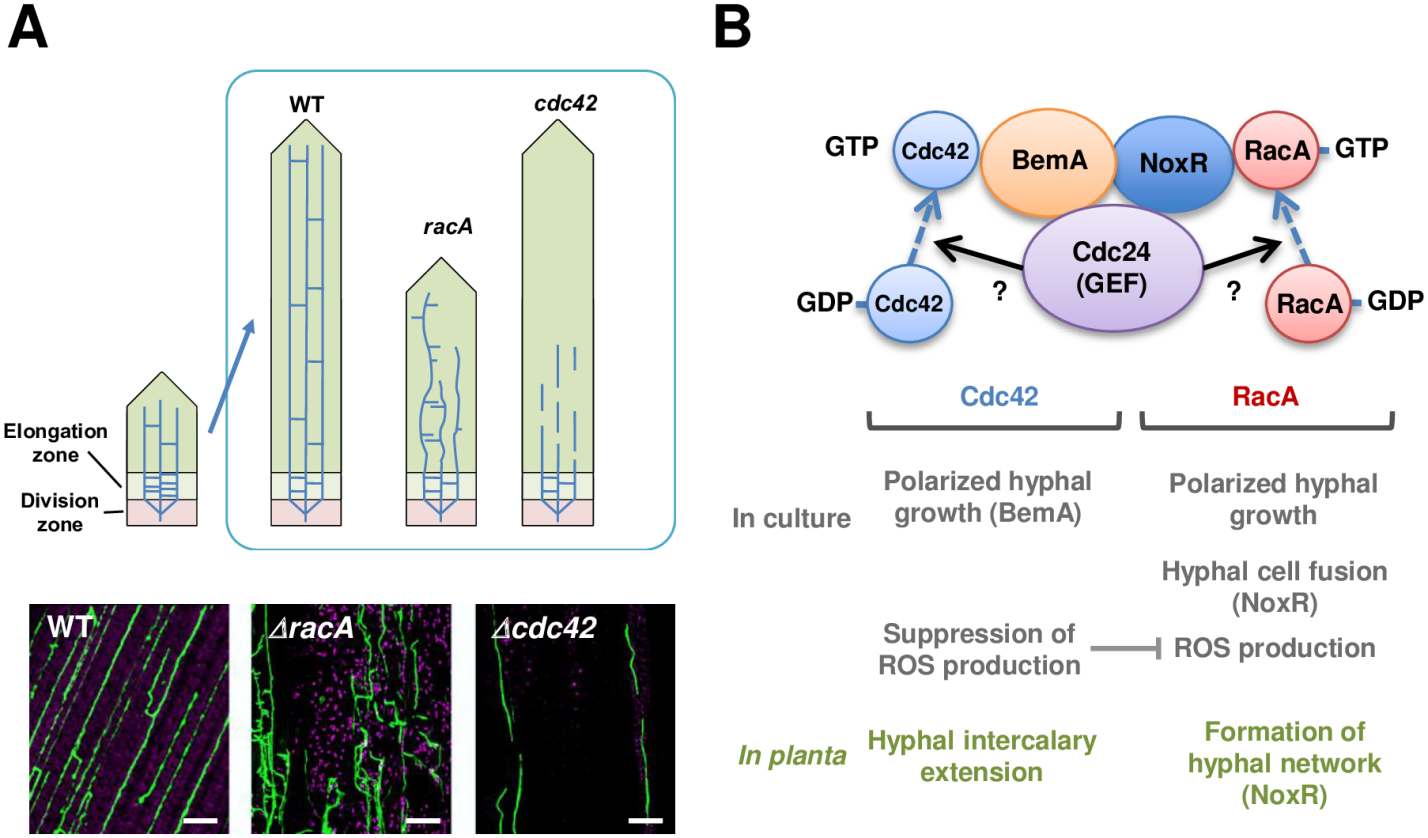
Summary of functions of *Epichloë festucae* Cdc42 and RacA. **(A)** Phenotype of *racA* and *cdc42* mutants indicated that RacA is involved in the regulation of growth pattern and cell-cell fusion of *E*. *festucae* hyphae, whereas Cdc42 is required for in intercalary hyphal growth in expanding host leaves. Hyphae of the endophyte in host plant are drawn as light blue lines in top diagram. Bars = 50 μm. **(B)** Summary of interactions between factors in Nox complex and functions of Cdc42 and RacA. Factors in parentheses indicate the interacting partner required for each function of small GTPases.

A large number of microbes can asymptomatically colonize the inside of plant tissues, but the mechanisms underlying the establishment of these symptomless infections are largely unknown. Key requirements for symptomless colonization by endophytic microbes must be; first, suppression or evasion of potential plant defense responses; and second, strict control of biomass and growth pattern in the host plant. In this study, we report that two highly homologous Rho GTPases, Cdc42 and RacA, have distinctive roles in hyphal growth of *E*. *festucae* in its grass host. RacA is required for maintaining a normal pattern of growth *in planta*, including growth parallel to the leaf axis of host tillers and formation of hyphal network via cell-cell fusion. On the other hand, Cdc42 is required for intercalary hyphal growth to establish systemic infection in expanding host leaves (Fig 12).

### Strict control of hyphal growth regulated by small GTPases is required for symbiotic infection of endophyte in grass plants

In the interaction between perennial ryegrass and wild type *E*. *festucae*, the majority of endophytic hyphae grow parallel to the growing axis of the host plant, with occasional branching and cell fusion to establish a network-like pattern of hyphae in the leaf (Fig 12A) [11, 14]. In the leaves, hyphae of wild type endophytes grow in the intercellular spaces [14, 15], and branched hyphae also tend to grow between the short sides of host cells. In contrast, the growth pattern of the *racA* mutant is clearly abnormal, including frequent branching and convolution of hyphae. Hyphae of some cell fusion mutants (e.g. *noxA*, *noxR* and *proA* mutants) were more abundant in the intercellular space than the wild type [15, 16, 20]. Infection of the *noxA* mutant resulted in enhanced expression of host defense genes [15]. Abnormal blebbing of the plant plasma membrane, adjacent to the multiple hyphae found in *noxR* mutant associations was frequently observed [16]. Observations such as these, indicate that unregulated growth of the cell fusion mutants in host plants may enhance plant stress responses, which is likely to be a cause of plant stunting. Thus, strict control of the growth pattern in the host plants is a key requirement for *E*. *festucae* to maintain a mutualistic interaction with the host.

While infection of ryegrass plants with the *cdc42* mutant caused no obvious host interaction phenotype, fragmentation of *cdc42* mutant hyphae was often observed in the lower parts of leaf tissues from the mature host plant, and colonization of the upper regions of mature leaf blades was rarely detected. In the smaller emerging leaves, systemic infection with the *cdc42* mutant was observed, suggesting that fragmentation of *cdc42* mutant hyphae may be caused as a result of host elongation. The growth rate of tillers of perennial ryegrass is approx. 1 cm/day [11], whereas the growth of wild type *E*. *festucae* strain Fl1 on PDA is approx. 3 mm/day. It thus follows, that the growth rate of *E*. *festucae* would have to be at least 3 times faster in host plants than in culture. It is proposed that hyphal cells of the endophyte are attached to plant cells by an extracellular matrix, and extend by intercalary growth *in planta*, rather than tip growth, the commonly observed pattern of growth for filamentous fungi growing in culture [11]. This mode of hyphal growth enables the endophyte to sustain the systemic infection in a fast-growing host plant. Examples of intercalary hyphal growth were reported in a wide range of fungal systems such as mushroom stipes, rhizomorphs, lichen thalli, asci and water mould hyphae [reviewed in 40, 41], but the mechanisms for intercalary hyphal growth are mostly unknown. Hyphal fragmentation of the *cdc42* mutant in the host plant suggests that Cdc42 is essential for intercalary hyphal growth. One of the known functions of Cdc42 is the regulation of intracellular vesicle transport [26]. In particular, Cdc42 controls the transport of vesicles near the site of the growing hyphal surface, by regulating the reorganization of the cytoskeleton. Therefore, Cdc42 may be involved in the transport of vesicles to the internal hyphal cells during intercalary extension of the hyphae in the host plant. During systemic infection, the phenotype of *cdc42* mutants could be partially recovered by the NoxR-binding form of Cdc42, as well as the BemA-binding form of RacA and even by additional expression of wild type RacA, which demonstrates that control of intercalary hyphal growth appears to be a common function among Cdc42 and RacA. However, the results suggest that Cdc42 plays the major role, since fragmentation of hyphae in the host plant is seldom observed for the *racA* mutant.

### Roles of RacA and Cdc42 in ROS production in *E*. *festucae*

While production of ROS was reduced in *racA* mutants, *cdc42* mutants had increased ROS production, which suggests that at least in culture, these Rho GTPases have opposite roles in the regulation of ROS production. In mammals, Rac is an essential positive regulator of Nox-mediated ROS production [42]. Cdc42 acts as a negative regulator of ROS production both in cell-free systems, as well as in Nox-expressing mammalian cell lines. Mammalian Cdc42 cannot activate Nox, but can bind to flavocytochrome *b*558 (composed of Nox2/p91^*phox*^ and p22^*phox*^) by an insertion domain similar to Rac, suggesting that Cdc42 may act as a negative regulator to maintain a low level of Nox activation by competing with Rac for binding with flavocytochrome *b*558 [43]. Likewise, *E*. *festucae* Cdc42 may compete with RacA for binding with the Nox complex composed of NoxA and Pro41 and/or NoxB and Pls1 [44–46]. Alternatively, Cdc42 and RacA could compete for the common activator of these small GTPases, which is Cdc24. Cdc24 is a guanine nucleotide exchange factor (GEF) which is known to activate both Cdc42 and RacA in various filamentous fungi [47, 48]. Therefore, loss of Cdc42 may enhance Cdc24 activation of RacA, leading to enhanced production of ROS in the *cdc42* mutant background.

### Distinctive and overlapping functions of Cdc42 and RacA in hyphal growth

The *racA* mutant demonstrated a severe growth defect in culture, including convoluted hyphae, as well as increased branching and septation. Although growth of the *cdc42* mutant on nutrient rich PDA was almost like wild type, irregular hyphal growth was also observed for *cdc42* mutants on nutrient poor water agar, suggesting that both RacA and Cdc42 are involved in the maintenance of hyphal polarity. Double knockout mutants of *racA* and *cdc42*, or knockout mutants of *cdc24* alone (probable common GEF for RacA and Cdc42) have never been isolated for *E*. *festucae* ([18] and this study). Consistently, *cdc24* mutants or *cdc42/racA* double mutants are lethal in *N*. *crassa*, *Aspergillus niger* and *A*. *nidulans*. [47, 49, 50], indicating that Cdc42 and RacA have overlapping function(s) essential for the viability (i.e. hyphal growth) of these fungi.

Interestingly, the width of hyphae was wider for the *E*. *festucae racA* mutant and thinner for *cdc42* mutant compared to wild type, suggesting that RacA and Cdc42 have distinct roles in hyphal growth. Distribution of actin patches was affected by a knockout of *racA* and *cdc42*. Actin patches, which are intracellular vesicles surrounded by F-actin, are considered to mediate endocytosis in fungal cells [51]. The altered distribution of actin patches in *cdc42* and *racA*, suggests there is a change of active vesicle trafficking near growing hyphal tips, which may affect the hyphal diameters of the mutants. Introduction of RacA-C12 (no binding with NoxR) into the *racA* mutant complemented the growth defect, which in turn suggests that RacA has another partner molecule, besides NoxR, for establishment of polarized hyphal growth. In contrast, RacA-C458 (BemA binding form), but not Cdc42-R8 (no binding with BemA), restored the hyphal growth defects of the *cdc42* mutant, indicating that binding to BemA is essential and sufficient for the regulation of hyphal polarity by Cdc42/RacA in culture.

As previously indicated by Harris [27], the contribution of RacA and Cdc42 to hyphal morphology is diverse among fungal species. Given that either *racA* or the *cdc42* mutant showed increased branching and convoluted hyphal growth in a number of species ([47, 49, 50] and this study), a shared function for RacA and Cdc42 is maintenance of hyphal polarized growth, in which both RacA and Cdc42 probably share common downstream effector(s). In contrast, it has been reported that *N*. *crassa* CDC-42 and RAC-1 have opposite roles in negative and positive chemotropism of conidial protrusions, namely germ tubes and conidial anastomosis tubes, respectively [52]. Endophyte Cdc42 and RacA have opposite roles in the regulation of ROS production. Such distinctive roles of Cdc42 and Rac could be determined by their interaction with specific downstream effectors.

### Functional differentiation between Cdc42 and RacA is partly determined by specific binding with components of the Nox complex

In this study, amino acid residues essential for specific binding of RacA to NoxR and Cdc42 to BemA were identified. Based on the predicted structure of both small GTPases, the binding site of RacA to NoxR locates to the exposed loop between the first helix and the second strand just in front of switch I, while the binding site of Cdc42 to BemA is located inside of switch II (See S10 Fig). Exchange of the GDP- to GTP-bound form induces a conformational change of switch I and II from being more flexible to becoming more ordered [28]. As the CA (GTP-bound)-form of RacA and Cdc42 localize to the hyphal tip and intracellular (vacuolar) membrane, binding of GTP to the small GTPases is probably essential for enhancing their interactions with NoxR and BemA, and other partner components at intracellular membranes.

Dissecting the roles of the multifunctional factors with several partner components, like RacA and Cdc42, is particularly difficult as a simple KO of the target genes causes complex phenotypes which result from consequential effects on many downstream factors. Domain swaps and amino acid substitutions were generated that enable Cdc42 to bind to NoxR and RacA to bind to BemA, allowing us to analyze the importance of their specific interactions. From our results, there are two outcomes when the NoxR-binding form of Cdc42 is introduced into the *racA* mutant: 1) No detectable recovery of the defect is observed in the *racA* mutant (e.g. polarized hyphal growth), or 2) The Phenotype of *racA* mutant is restored to wild type (e.g. hyphal fusion, symbiotic infection). The explanations for these outcomes are: 1) RacA has another partner protein besides NoxR, to regulate the function or 2) NoxR is the essential and sufficient downstream regulator for the function. Similarly, there are three observed outcomes for complementation of the *cdc42* mutant with the BemA-binding form of RacA; 1) No detectable recovery of the defect (e.g. colony growth), 2) phenotype is recovered, resembling the wild type (i.e. polarized hyphal growth) or 3) The phenotype is partially recovered and similar to wild type (intercalary hyphal growth *in planta*). These outcomes are explained with: 1) Cdc42 has another partner molecule to regulate the function, 2) BemA is the essential and sufficient downstream regulator of Cdc42 function, and 3) Both Cdc42 and RacA are co-regulators of the function. Fig 12B summarizes the distinct and overlapping functions of *E*. *festucae* RacA and Cdc42. The importance of small GTPase partner proteins (i.e. NoxR and BemA) for each function can be determined by the approach used in this study, but it should be taken into account that the introduced mutation may affect the interaction of these small GTPases with other, unidentified downstream components. Determining possible effects on downstream components requires further investigation.

The small GTPases Cdc42 and Rac are highly conserved in eukaryotes. The core downstream effectors for Cdc42 and Rac in mammals can be categorized into three distinct classes, namely protein kinases (e.g. PAKs and MEKK), actin binding proteins (i.e. WASPs, WAVEs and IQGAPs), and lipid modifying enzymes (e.g. PI3K, PLC and PLD) [53]. Homologues of these effectors can be identified in fungal genomes, thus some of them represent potential downstream effectors of fungal Cdc42 and Rac. Many of these downstream effectors interact with and are co-regulated by both Cdc42 and RacA via binding to the CRIB (Cdc42/Rac interactive binding) domain [54], which can be found in Cdc42/Rac binding proteins in fungi [52]. There are also several mammalian downstream effectors, which specifically bind to either Rac (e.g. PIP5 Kinase, p67^phox^) or Cdc42 (e.g. MRC Kinase, DRF3) [53], but most fungal homologues of such Rac- and/or Cdc42-interacting factors have not been extensively analyzed.

The multi-functional nature of fungal Cdc42 and RacA, which is presumably caused by the sharing of multiple downstream effectors, requires further analysis, as an understanding of this functional network may elucidate how the activation of various cellular events are activated in a controlled fashion.

## Materials and methods

### Biological material, growth conditions and inoculation

*Epichloë festucae* strains (S2 Table) were grown on potato dextrose agar (PDA) or water agar (3% agar) at 23°C. Inoculation of endophyte-free seedlings of perennial ryegrass (*Lolium perenne* cv. Yatsukaze) with *E*. *festucae* was performed by the method of Latch and Christensen [55]. Inoculated plants were grown as previously described [56].

### DNA preparations and hybridizations

Fungal genomic DNA was isolated from mycelium as previously described [57] or using an Extract-N-Amp plant PCR kit (Sigma). Genomic digests were transferred to positively charged nylon membranes (Hybond-N^+^, GE healthcare) by capillary transfer and fixed by UV cross-linking in a UV cross-linker (CL-1000, Ultra-Violet Products) at 7 x 10^4^ μJ/cm^2^. Filters were probed with knockout vector pNPP50 labeled with [α-^32^P] dCTP (3000 Ci/mmol; Perkin Elmer) using a random-primed DNA labeling kit (Megaprime DNA Labelling System, GE Healthcare). Hybridizations were performed at 42°C for 20 h in 5 x SSPE (20x SSPE; 3 M NaCl, 173 mM NaH_2_PO_4_-2H_2_O, 25 mM EDTA), 50% formamide, 5x Denhardt’s solution, 1% SDS and 100 μg/ml denatured salmon sperm DNA. Membranes were washed with 1 x SSPE and 0.2% SDS at 65°C for 10 min and then with 0.1 x SSPE and 0.1% SDS at 65°C for 5 min. The membrane was then subjected to autoradiography.

### Construction of vectors for deletion, complementation and overexpression of *E*. *festucae* genes, and yeast two-hybrid assay

Standard PCR amplifications of genomic and plasmid DNA templates were performed using PrimeStar HS DNA polymerase (Takara) or GoTaq Master Mix (Promega). Vectors for gene knock out and gene expression in *E*. *festucae* used in this study are listed in S3 Table. Sequences of primers used for construction of vectors for gene knock out and expression in *E*. *festucae* are listed in S4 Table. Vectors for yeast two hybrid assay used in this study are listed in S5 Table. Primers used for the construction of vectors for yeast two hybrid assay are listed in S6 Table.

### Yeast two-hybrid assay

Yeast two-hybrid assays using pGADT7 or pGBKT7 (Clontech) based constructs were performed according to the manufacturer’s instructions (MATCHMAKER Two-Hybrid System3, Clontech). Yeast strain AH109 was transformed with prey (pGADT7 derivatives) and bait (pGBKT7 derivatives) vectors (S5 Table) using *S*. *cerevisiae* Direct Transformation Kit (Wako). Transformed yeast strains (S7 Table) were selected on SD medium lacking leucine and tryptophan (-L/-T). Transformants were plated on SD medium lacking leucine and tryptophan (-L/-T) or lacking leucine, or lacking leucine, tryptophan, histidine and adenine (-L/-T/-H/-A). Growth on the latter indicates an interaction between bait and prey.

### *E*. *festucae* transformation

Protoplasts of *E*. *festucae* were prepared as described previously [58]. Protoplasts were transformed with 5 μg of either circular or linear (for gene KO) plasmids using the method described previously [59]. In case of co-transformation of multiple vectors for BiFC assay, 7.5 μg of each vector was used. Vectors used for *E*. *festucae* transformation were listed in S3 Table. Transformants were selected on PDA containing either hygromycin (150 μg/ml), geneticin (400 μg/mL) or both antibiotics for co-transformation.

### Microscopy

Images of GFP-labelled or aniline blue/WGA-AF488-stained *E*. *festucae* strains in host plant, GFP-tagged proteins in *E*. *festucae* hyphae and *E*. *festucae* hyphae stained with Calcofluor white (Fluka) were captured using a confocal laser scanning microscope FV1000-D (Olympus) or a BX51 fluorescence microscope (Olympus). The laser for detection of GFP and AF488 fluorescence was used as the excitation source at 488 nm, and GFP fluorescence was recorded between 515 and 545 nm. The laser for detection of Calcofluor white and aniline blue was used as the excitation source at 405 nm, and fluorescence was recorded between 425 nm and 475 nm. The numbers of hyphal cell fusions were counted under the microscope from at least 30 sites as previously reported [13]. Aniline blue/WGA-AF488 staining was performed as previously described [18].

### Detection of superoxide

The relative intensity of ROS generation was determined by counting photons from L-012-mediated chemiluminescence as reported previously [60]. L-012 (Wako) is a luminol derivative that is highly sensitive to superoxide radicals. To detect the ROS production in *E*. *festucae* hyphae, 0.5 mM L-012 was added to 9 mm^2^ colony blocks grown for 12 days on PDA. Chemiluminescence was monitored continuously using a photon image processor equipped with a sensitive CCD camera in a dark chamber at 20°C (Aquacosmos 2.5; Hamamatsu Photonics), and quantified using the U7501 program (Hamamatsu Photonics).

### Preparation of DNA and quantitative PCR

Extraction of genomic DNA of perennial ryegrass, infected with endophyte strains was performed using Extract-N-Amp Plant PCR kit (Sigma). Ten mg of plant tissue was ground using an electric homogenizer (BioMasher, Nippi), suspended in 150 μl of extraction buffer and centrifuged at 12,000 xg for 1 min. Total DNA was purified from the supernatant (100 μl) according the manufacturer’s instructions. Quantitative PCR was performed using LightCycler Quick System 350S (Roche Applied Science) with Thunderbird SYBR qPCR Mix (Toyobo). Gene-specific primers used for expression analysis are listed in S4 Table.

### DNA sequencing and bioinformatics

DNA fragments were sequenced by the dideoxynucleotide chain termination method using Big-Dye (version 3) chemistry (Applied Biosystems). Products were separated on an ABI 3130 analyzer (Applied Biosystems). Sequence data was analyzed and annotated in MacVector (version 14.5 or earlier; MacVector Inc.). Three-dimensional structure of *E*. *festucae* small GTPases were predicted by Iterative threading assembly refinement (I-TASSER) [61] with the template library updated on 17 Mar 2017, and drawn using Swiss-PdbViewer [62].

### Phylogenetic analysis

The deduced protein sequences of fungal small GTPases (S1 Table) were aligned using the ClustalW program [63] with default settings. Phylogenetic analysis was conducted using the neighbor-joining method [64] using MEGA ver. 6.06 [65] with 1000 bootstrap trials.

### Accession numbers

Sequence data from this article can be found in the GenBank database under the accession numbers LC228222 (*E*. *festucae cdc42*), LC228223 (*E*. *festucae rhoA*), LC228224 (*E*. *festucae rhoB*), LC228225 (*E*. *festucae rhoC*) and LC228226 (*E*. *festucae rhoD*).

## Supporting information

S1 Fig Phylogenetic analysis of small GTPase from *E*. *festucae* and Ascomycota fungi. The tree was prepared by the neighbor-joining method [64] using MEGA ver. 6.06 [65]. The scale bar corresponds to 5 estimated amino acid substitutions per site. Numbers at the nodes indicate the percentage of 1000 bootstrap replicates that supported each labeled interior branch. Ef; *Epichloë festucae*, Fg; *Fusarium graminearum*, Mo; *Magnaporthe oryzae*, Nc; *Neurospora crassa*.
(PDF)

S2 Fig Interactions of *E*. *festucae* Rho GTPases, RacA and Cdc42, with components of fungal Nox complex. Yeast strain AH109 was transformed with prey and bait vector as indicated and plated on to SD medium lacking leucine and tryptophan (-L/-T) or lacking leucine, tryptophan, histidine and adenine (-L/-T/-H/-A). Growth on the latter indicates an interaction between bait and prey. Rho GTPases have mutation in C-terminal plasma membrane localization signal. **(A)** Yeast two-hybrid assays of the interactions between truncated BemA and Cdc42. Domain structure of full length and truncated BemA used for yeast two-hybrid assays are indicated at the left side. **(B)** Yeast two-hybrid assays of the interactions between *E*. *festucae* NoxR, BemA, Cdc24, Cdc42 and RacA.
(PDF)

S3 Fig Expression of constitutively active form of Cdc42 (CA-Cdc42) or RacA (CA-RacA) induces swelling of *E*. *festucae* hyphae. CA-RacA or CA-Cdc42 were expressed in *E*. *festucae* wild type, *noxR* mutant (*ΔnoxR*) or *bemA* (*ΔbemA*) mutant under the control of the Tef promoter. Hyphae of endophyte strains were stained with Calcofluor white and monitored with confocal laser microscopy. Bars = 10 μm.
(PDF)

S4 Fig Targeted gene replacement of the *E*. *festucae cdc42* locus. **(A)** Physical map of the *cdc42* wild-type (WT) genomic region and linear insert of *Cdc42* replacement construct, showing restriction enzyme sites for *Eco*RV (EV), *Eco*RI (EI), *Bgl*II (Bg) and *Nde*I (N). The mutated genomic locus of *cdc42* deletion mutant (*Δcdc42*) is depicted to show homologous recombination of the *hph* cassette. Primers used for screening for the replacement event are indicated by arrowheads. **(B)** Southern blot analysis of WT and *cdc42* mutant. *Nde*I genomic digests of WT and *cdc42* mutant strains. were proved with [^32^P]-labelled pNPP50.
(PDF)

S5 Fig Comparison of hyphal volume per compartment between wild type, *cdc42* and *racA* mutants. Hyphae of endophyte strains were stained with calcofluor white and monitored with confocal laser microscopy. Bars = 20 μm. Area of cell compartments for each strain was measured using ImageJ software. Data are means ± standard error. n = 20. Data marked with asterisks are significantly different from wild type as assessed by two-tailed Student’s *t* tests: *P < 0.05, **P < 0.01.
(PDF)

S6 Fig Hyphal growth of *E*. *festucae cdc42* and *racA* mutants on PDA. *E*. *festucae* wild type (WT), *cdc42* and *racA* mutants were grown on PDA for 14 days. Hyphae of endophyte strains were stained with Calcofluor white and monitored with confocal laser microscopy. Bars = 30 μm.
(PDF)

S7 Fig Distribution of actin patches in hyphal tip of *E*. *festucae* wild type, *cdc42* and *racA* mutants. Subcellular localization of actin patches visualized by Lifeact-GFP in hyphae of *E*. *festucae* wild type (WT), *cdc42* (*Δcdc42*) and *racA* (*ΔracA*) mutants after growth on PDA for 11 days. Arrowheads indicate accumulation of actin patches near hyphal tips. Bars = 5 μm.
(PDF)

S8 Fig ROS production in colony of *E*. *festucae*. Hyphae of endophyte were grown on PDA for 12 days and O_2_^-^ production of centeral, middle or growing edge (tip) part of colony was detected as L-012 mediated chemiluminescence. Chemiluminescence images were obtained using CCD camera. Data are means ± standard devision 15 sites from 5 colonies of each strain. Data marked with asterisks are significantly different as assessed by two-tailed Student’s *t* tests: **P < 0.01.
(PDF)

S9 Fig Growth of perennial ryegrass infected with wild type *E*. *festucae*, *racA* mutant or *cdc42* mutant. Photographs were taken approx. 2 months after inoculation. Bars = 10 cm.
(PDF)

S10 Fig Three-dimensional structure models of *E*. *festucae* Cdc42 and RacA. Three-dimensional structure of Rho GTPases are predicted by iterative threading assembly refinement (I-TASSER [61]). Amino acid residues required for specific binding between Cdc42 and BemA, or RacA and NoxR are indicated blue and red letters, respectively. Conf. Score; Confidence score, Sol. Acces.; Predicted solvent accessibility.
(PDF)

S11 Fig Alignment of deduced amino acid sequences of *E*. *festucae* and fungal Cdc42 and Rac. Amino acids specifically conserved among fungal Cdc42 and RacA are indicated by blue and red letters, respectively. An; *Aspergillus nidulans* (strain FGSC A4), Bc; *Botrytis cinerea* (T4), Bs; *Bipolaris sorokiniana* (ND90Pr), Co; *Colletotrichum orbiculare* (MAFF 240422), Cp; *Claviceps purpurea* (20.1), Ct; *Colletotrichum trifolii* (race 1), Fg; *Fusarium graminearum* (PH-1), Fo; *Fusarium oxysporum* f. sp. *cubense* (race 1), Mg; *Magnaporthe grisea* (70–15), Nc; *Neurospora crassa* (OR74A), Pd; *Penicillium digitatum* (PHI26), Sr; *Sporisorium reilianum* (SRZ2), Tm; *Talaromyces marneffei* (ATCC 18224), Um; *Ustilago maydis* (Bub8).
(PDF)

S12 Fig Identification of essential amino acids for specific interactions between Cdc42 and BemA or RacA and NoxR. **(A)** Yeast two-hybrid assays of the interactions between *E*. *festucae* NoxR and mutated RacA. **(B)** Yeast two-hybrid assays of the interactions between *E*. *festucae* BemA and chimeric or mutated Cdc42 and RacA. Rho GTPases have mutation in C-terminal plasma membrane localization signal. Yeast strain AH109 was transformed with prey and baid vector as indicated and plated on to SD medium lacking leucine and tryptophan (-L/-T) or lacking leucine, tryptophan, histidine and adenine (-L/-T/-H/-A). Growth on the latter indicates an interaction between bait and prey.
(PDF)

S13 Fig Complementation of *racA* mutant by *RacA* under control of the TEF promoter. **(A)** Colony morphology and diameter of E. festucae wild type (WT), *racA* mutant and complemented strains grown on PDA for 12 days. Data are means ± standard error. n = 3. Data marked with asterisks are significantly different from wild type as assessed by two-tailed Student’s *t* tests: **P < 0.01. **(B)** Hyphal growth of WT, *racA* mutant and complemented strains on water agar. *E*. *festucae* strains were stained with calcofluor white and monitored with confocal laser microscopy. Arrowheads indicate hyphal fusions. Bars = 10 μm. **(C)** L-012-mediated detection of ROS production by *E*. *festucae* WT, *racA* mutant and complemented strains. Colony edge of endophyte strains grown on PDA was treated with L-012 and ROS production was detected as chemiluminescence. Value of chemiluminescence relative to wild type was scored. Data are means ± standard error. n = 5. Data marked with asterisks are significantly different from wild type as assessed by two-tailed Student’s *t* tests: **P < 0.01. **(D)** Phenotype of perennial ryegrass infected with *E*. *festucae* WT, *racA* mutant or complemented strains. Photographs were taken approx. 8 weeks after inoculation.
(PDF)

S14 Fig Hyphal growth and symbiotic infection of *E*. *festucae* wild type, *cdc42* and complemented strains. **(A)** *E*. *festucae* strains were grown on 3%water agar for 10 days. Hyphae of endophyte strains were stained with calcofluor white and monitored with confocal laser microscopy. Bars = 30 μm. **(B)** Colonization of *E*. *festucae* WT, *cdc42* mutant and complemented strains in top part of perennial ryegrass tillers approx. 2 months after inoculation. Hyphae (blue lines) and septa (green dots) were visualized by WG-AF488/aniline blue staining monitored by confocal microscopy. Bars = 40 μm.
(PDF)

S1 Table Small GTPases of Ascomycota fungi, *E*. *festucae*, *N*. *crassa*, *F*. *graminearum* and *M*. *oryzae*. (XLSX)

S2 Table Fungal strains used in this study. (XLSX)

S3 Table Plasmids for gene knock out and expression in *E*. *festucae* used in this study. (XLSX)

S4 Table Primers for sequencing, vector construction and quantitative PCR used in this study. (XLS)

S5 Table Plasmids for yeast two-hybrid assay used in this study. (XLSX)

S6 Table Primers for construction of vectors for yeast two-hybrid assay. (XLS)

S7 Table Yeast strains used in this study. (XLSX)
