# Supplementary figures and images for: Two closely related Rho GTPases, Cdc42 and RacA, of the en-dophytic fungus Epichloë festucae have contrasting roles for ROS production and symbiotic infection synchronized with the host plant
Source: PLoS Pathog. 2018 Jan 25;14(1):e1006840. doi: 10.1371/journal.ppat.1006840 (PMC5785021; doi:10.1371/journal.ppat.1006840)

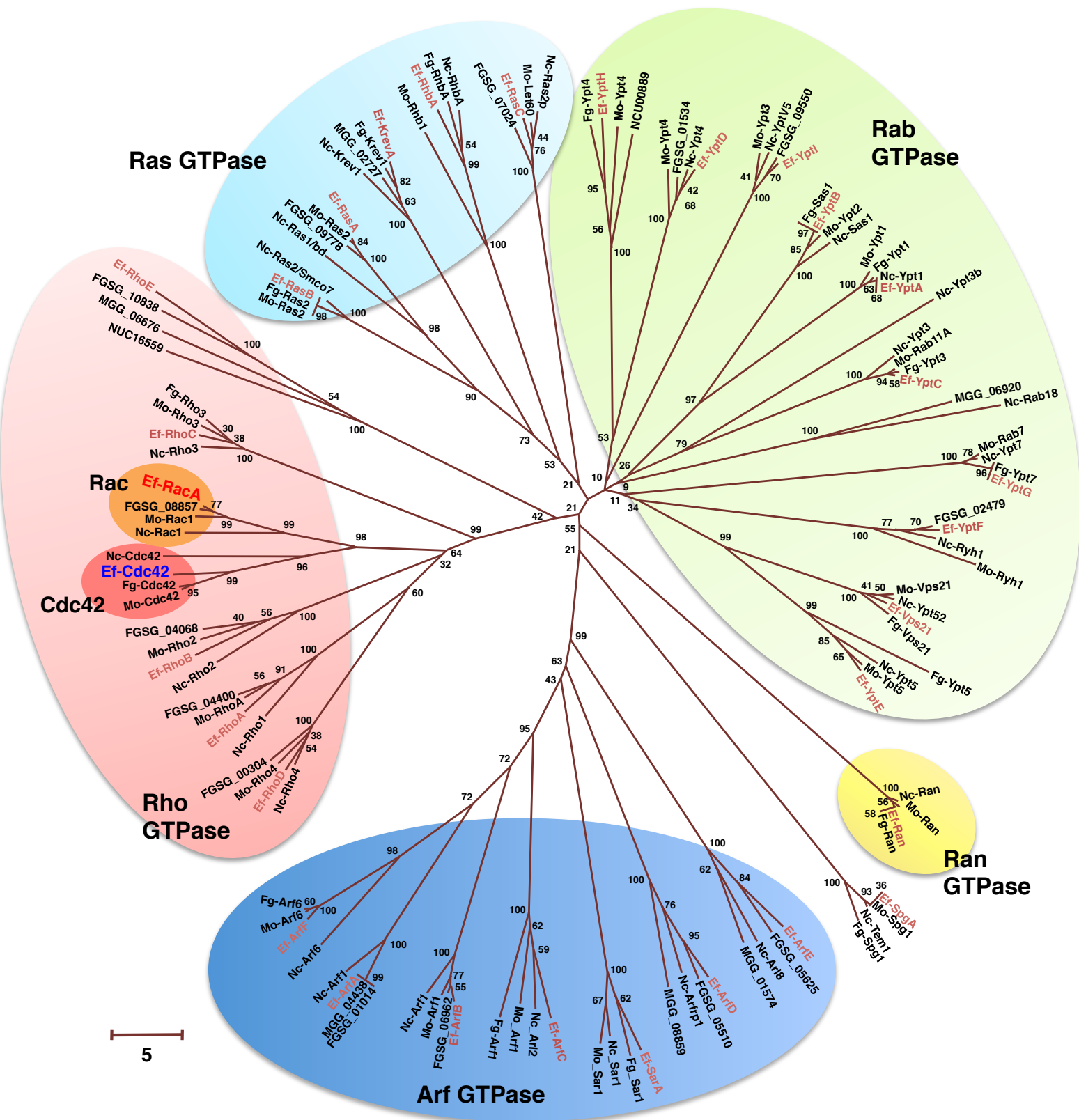

Supplement: S1 Fig — The tree was prepared by the neighbor-joining method [64] using MEGA ver. 6.06 [65]. The scale bar corresponds to 5 estimated amino acid substitutions per site. Numbers at the nodes indicate the percentage of 1000 bootstrap replicates that supported each labeled interior branch. Ef; Epichloë festucae, Fg; Fusarium graminearum, Mo; Magnaporthe oryzae, Nc; Neurospora crassa. (PDF) [file ppat.1006840.s001.pdf]

# A

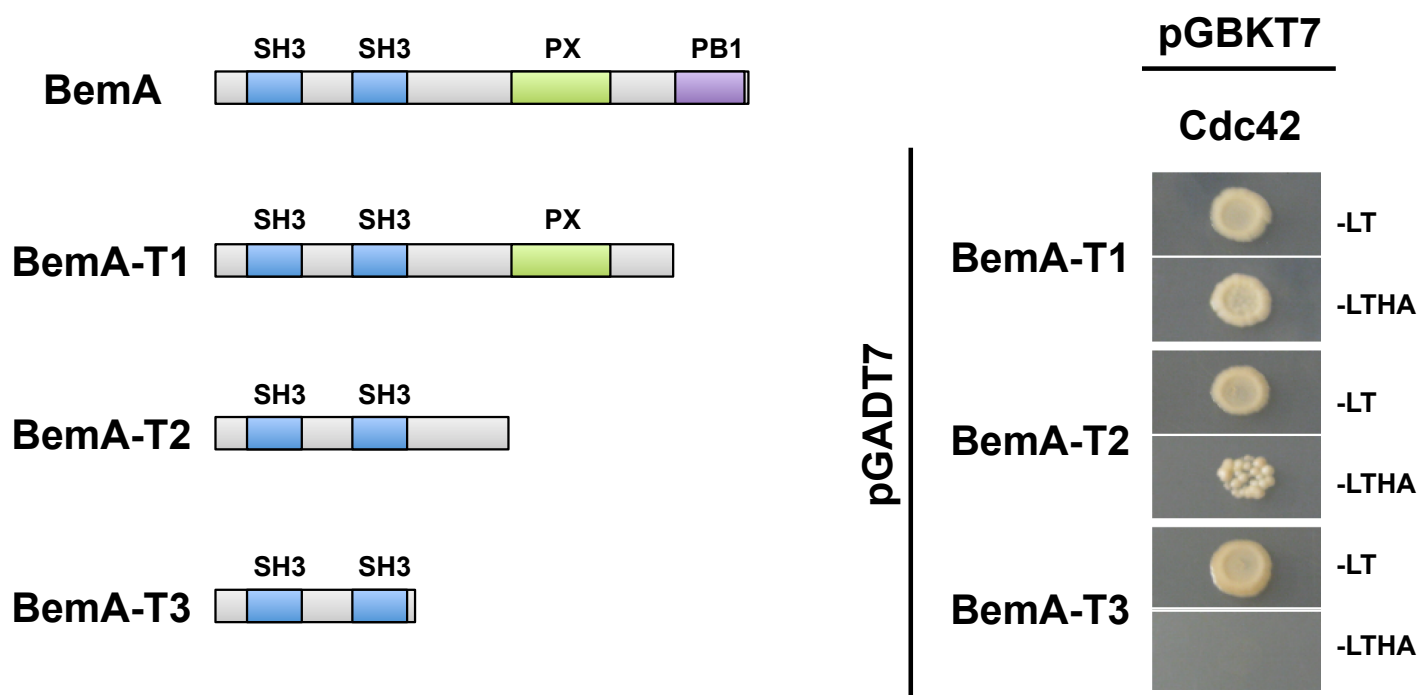

# B

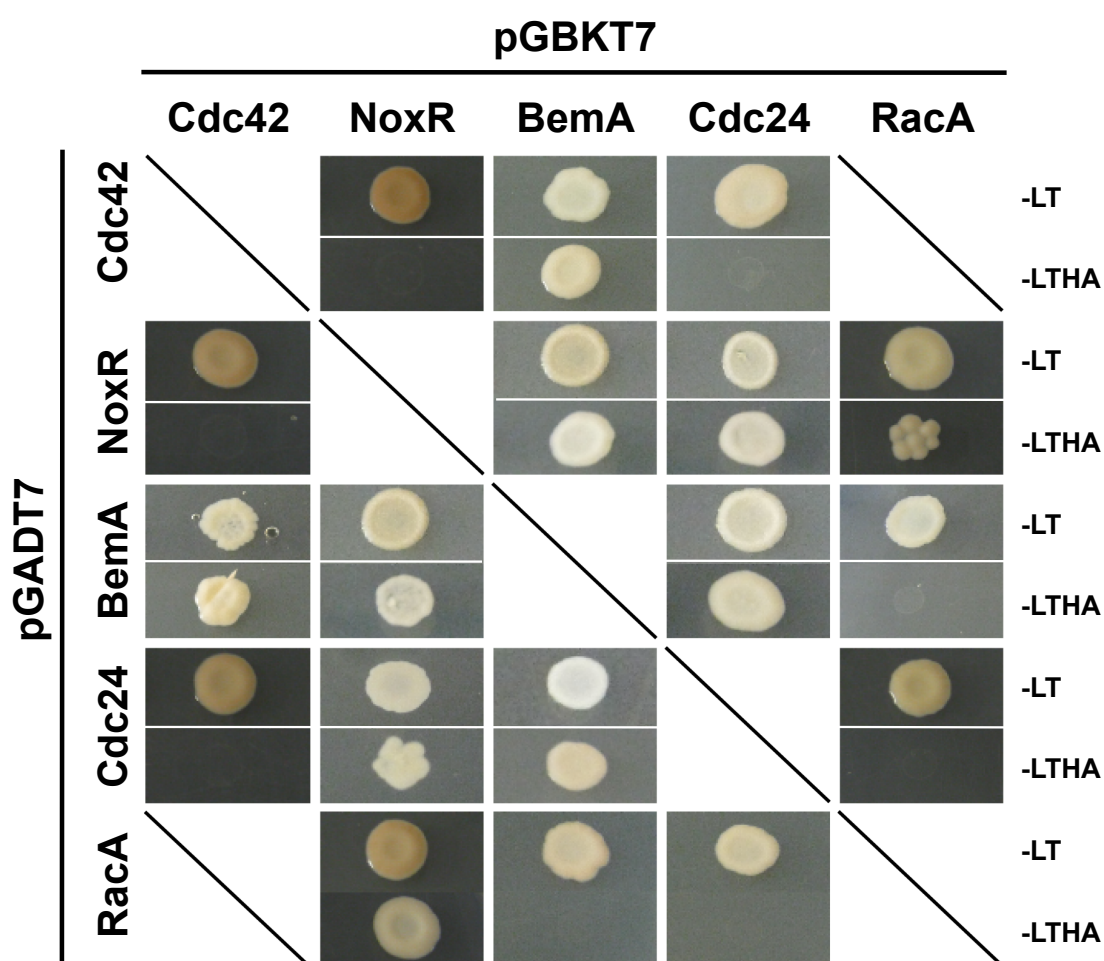

Supplement: S2 Fig — Yeast strain AH109 was transformed with prey and bait vector as indicated and plated on to SD medium lacking leucine and tryptophan (-L/-T) or lacking leucine, tryptophan, histidine and adenine (-L/-T/-H/-A). Growth on the latter indicates an interaction between bait and prey. Rho GTPases have mutation in C-terminal plasma membrane localization signal. (A) Yeast two-hybrid assays of the interactions between truncated BemA and Cdc42. Domain structure of full length and truncated BemA used for yeast two-hybrid assays are indicated at the left side. (B) Yeast two-hybrid assays of the interactions between E. festucae NoxR, BemA, Cdc24, Cdc42 and RacA. (PDF) [file ppat.1006840.s002.pdf]

**WT/CA-Cdc42**

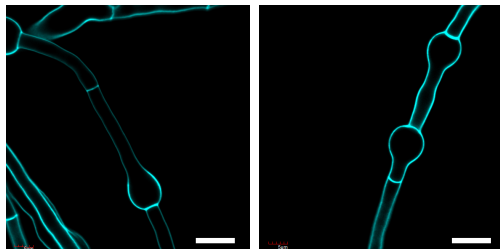

**WT/CA-RacA**

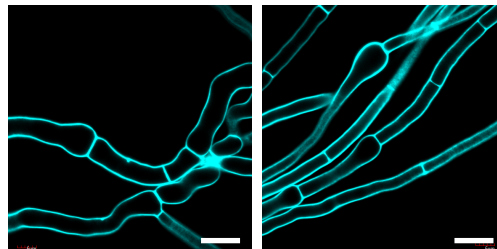

**$\Delta noxR$ /CA-Cdc42**

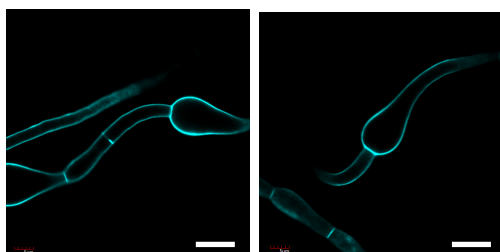

**$\Delta noxR$ /CA-RacA**

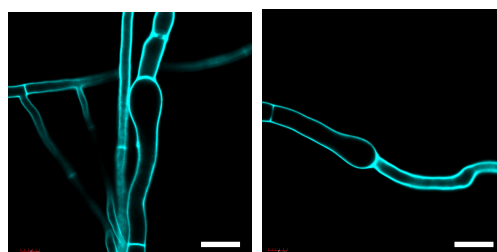

**$\Delta bemA$ /CA-Cdc42**

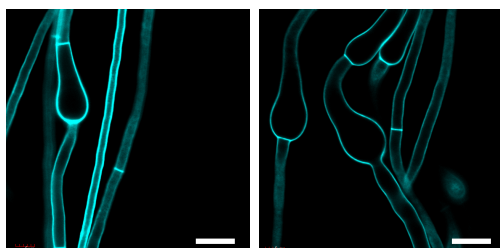

**$\Delta bemA$ /CA-RacA**

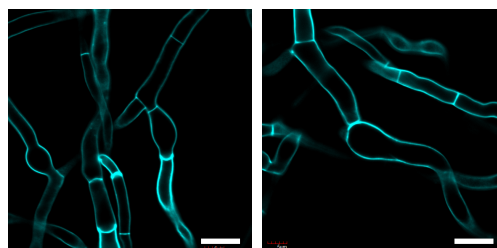

Supplement: S3 Fig — CA-RacA or CA-Cdc42 were expressed in E. festucae wild type, noxR mutant (ΔnoxR) or bemA (ΔbemA) mutant under the control of the Tef promoter. Hyphae of endophyte strains were stained with Calcofluor white and monitored with confocal laser microscopy. Bars = 10 μm. (PDF) [file ppat.1006840.s003.pdf]

**A**

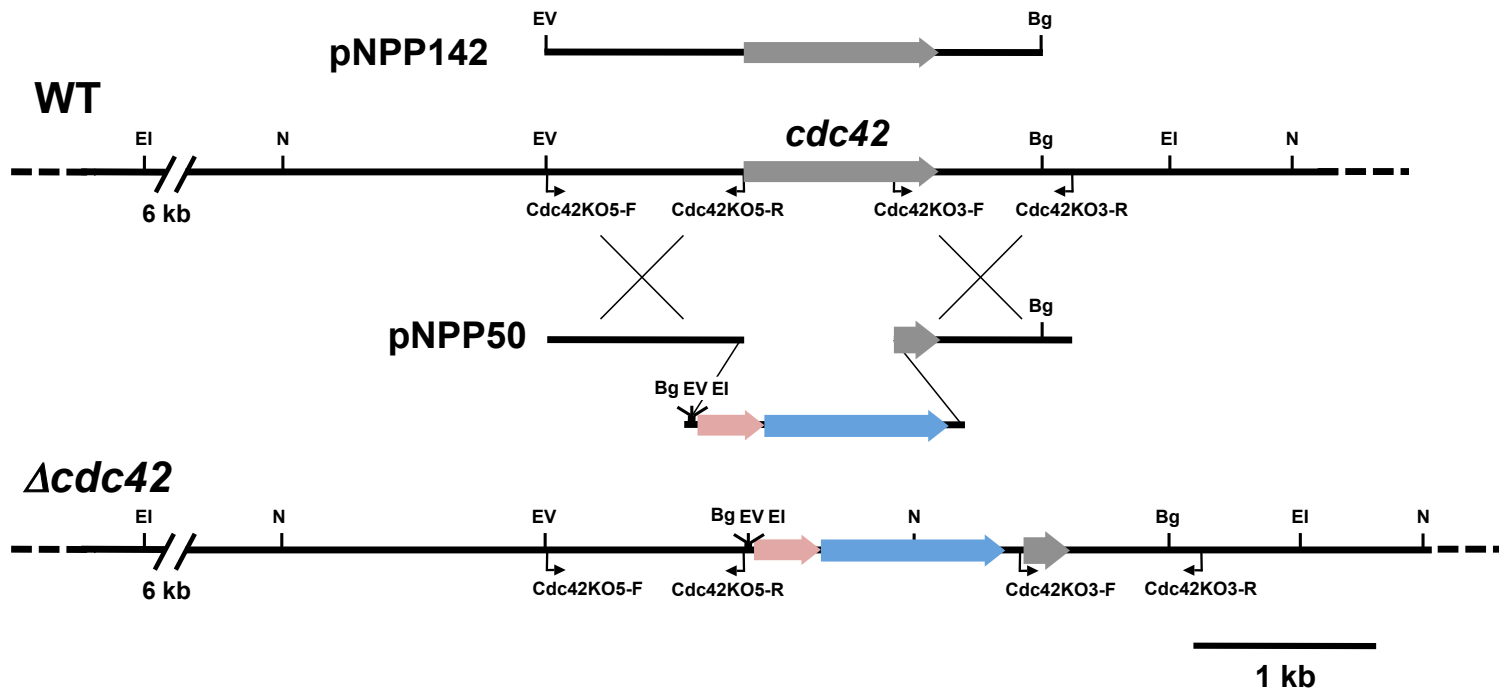

**B**

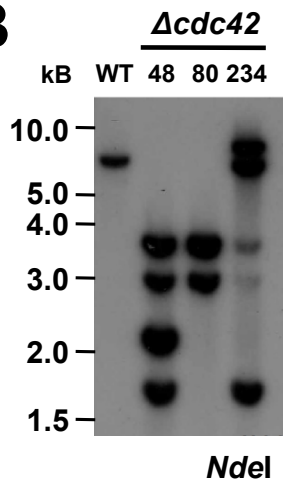

Supplement: S4 Fig — (A) Physical map of the cdc42 wild-type (WT) genomic region and linear insert of Cdc42 replacement construct, showing restriction enzyme sites for EcoRV (EV), EcoRI (EI), BglII (Bg) and NdeI (N). The mutated genomic locus of cdc42 deletion mutant (Δcdc42) is depicted to show homologous recombination of the hph cassette. Primers used for screening for the replacement event are indicated by arrowheads. (B) Southern blot analysis of WT and cdc42 mutant. NdeI genomic digests of WT and cdc42 mutant strains. were proved with [32P]-labelled pNPP50. (PDF) [file ppat.1006840.s004.pdf]

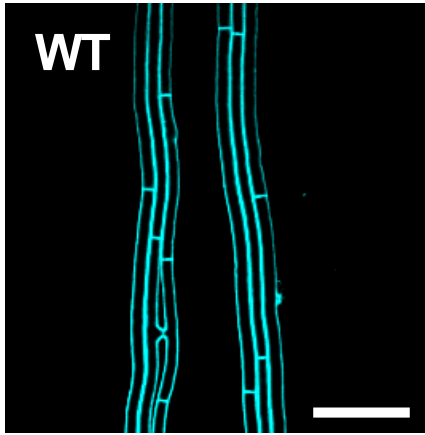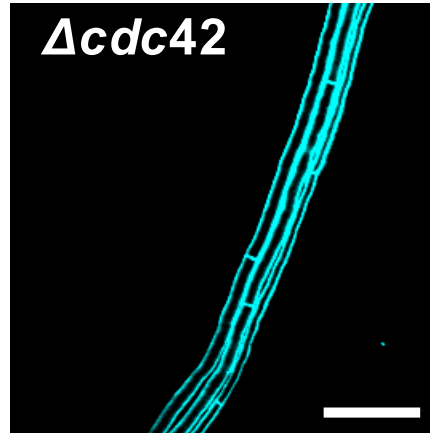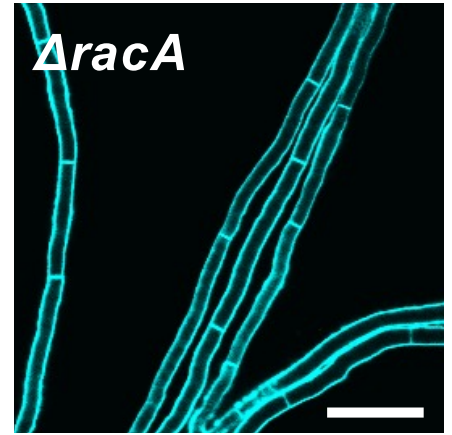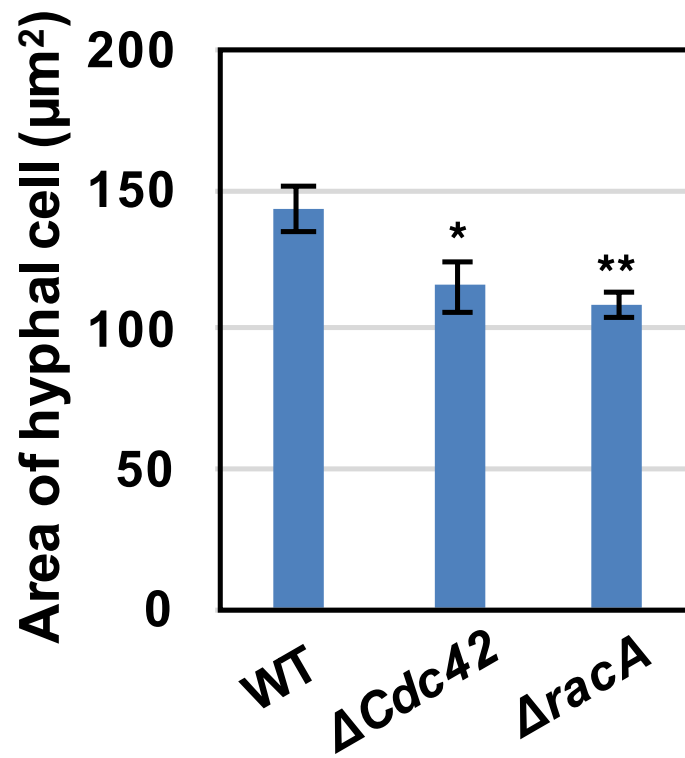

Supplement: S5 Fig — Hyphae of endophyte strains were stained with calcofluor white and monitored with confocal laser microscopy. Bars = 20 μm. Area of cell compartments for each strain was measured using ImageJ software. Data are means ± standard error. n = 20. Data marked with asterisks are significantly different from wild type as assessed by two-tailed Student’s t tests: *P < 0.05, **P < 0.01. (PDF) [file ppat.1006840.s005.pdf]

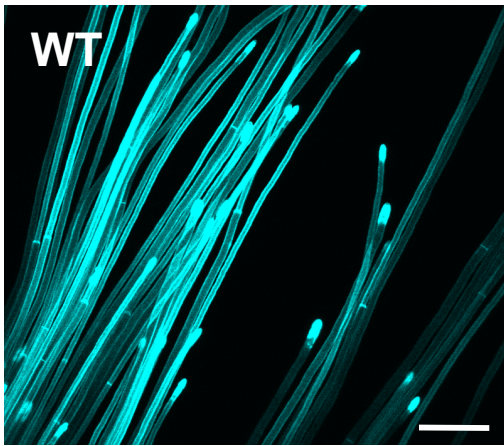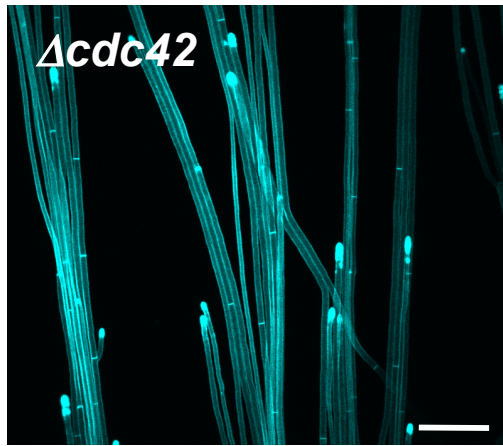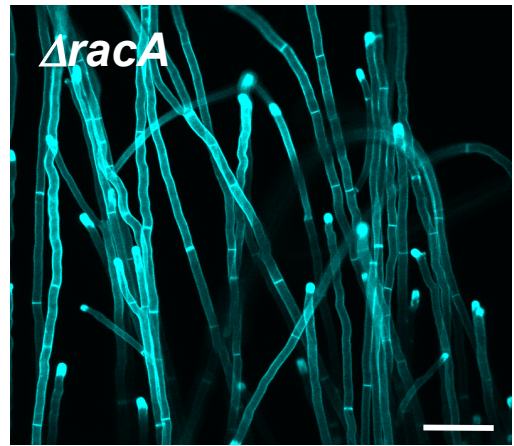

Supplement: S6 Fig — E. festucae wild type (WT), cdc42 and racA mutants were grown on PDA for 14 days. Hyphae of endophyte strains were stained with Calcofluor white and monitored with confocal laser microscopy. Bars = 30 μm. (PDF) [file ppat.1006840.s006.pdf]

**WT**

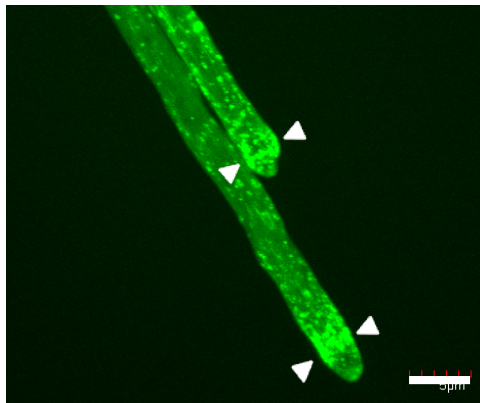

**$\Delta cdc42$**

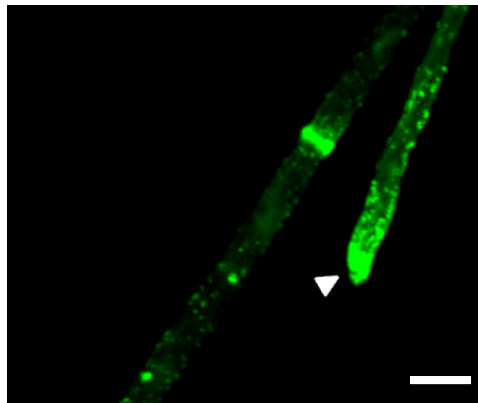

**$\Delta racA$**

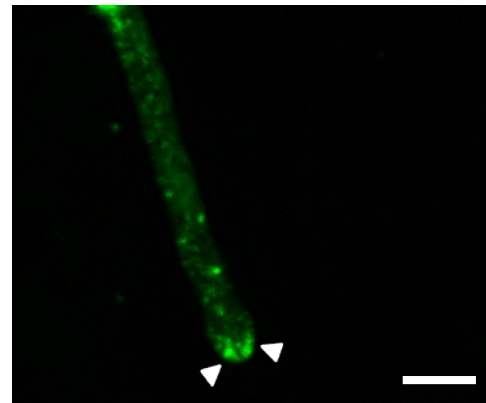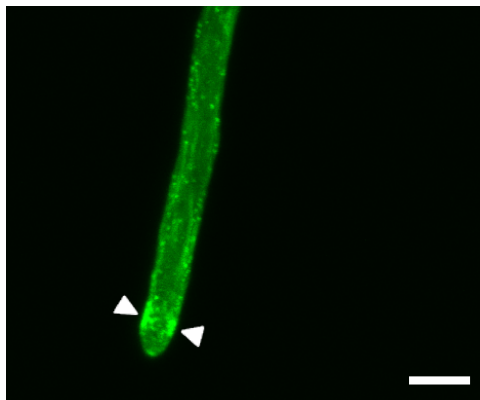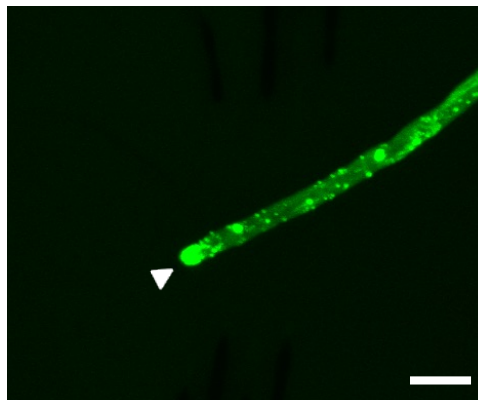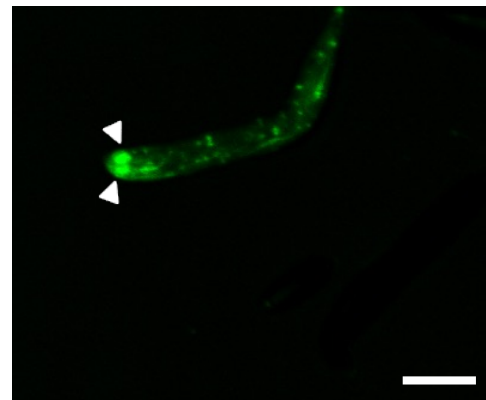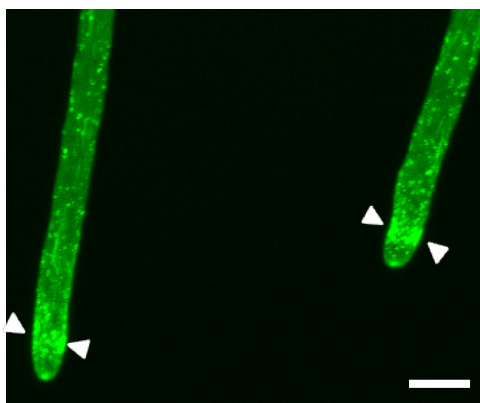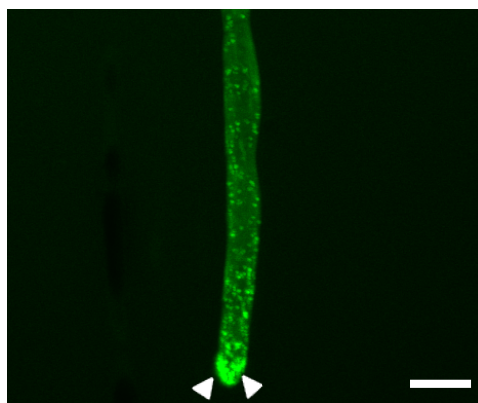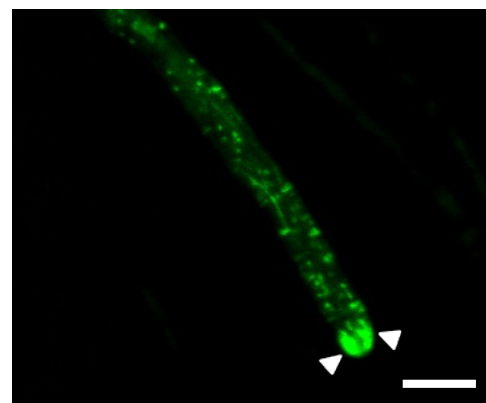

Supplement: S7 Fig — Subcellular localization of actin patches visualized by Lifeact-GFP in hyphae of E. festucae wild type (WT), cdc42 (Δcdc42) and racA (ΔracA) mutants after growth on PDA for 11 days. Arrowheads indicate accumulation of actin patches near hyphal tips. Bars = 5 μm. (PDF) [file ppat.1006840.s007.pdf]

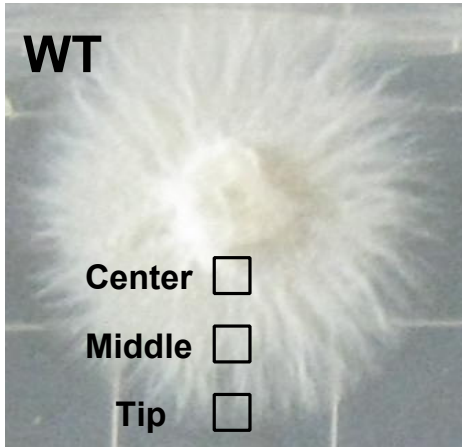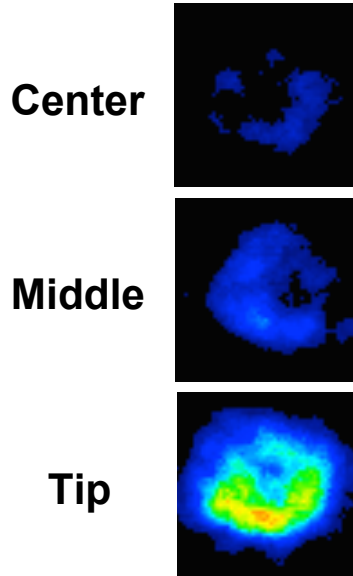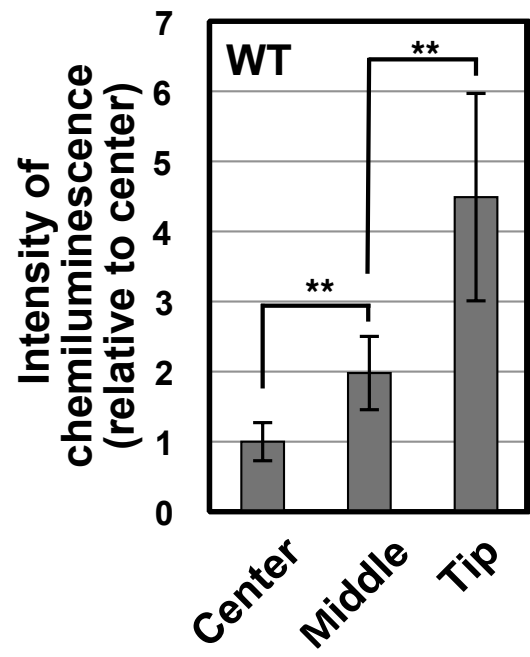

Supplement: S8 Fig — Hyphae of endophyte were grown on PDA for 12 days and O2- production of centeral, middle or growing edge (tip) part of colony was detected as L-012 mediated chemiluminescence. Chemiluminescence images were obtained using CCD camera. Data are means ± standard devision 15 sites from 5 colonies of each strain. Data marked with asterisks are significantly different as assessed by two-tailed Student’s t tests: **P < 0.01. (PDF) [file ppat.1006840.s008.pdf]

**WT**

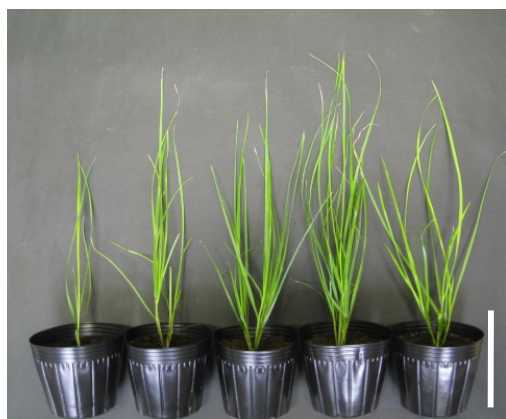

**$\Delta racA$**

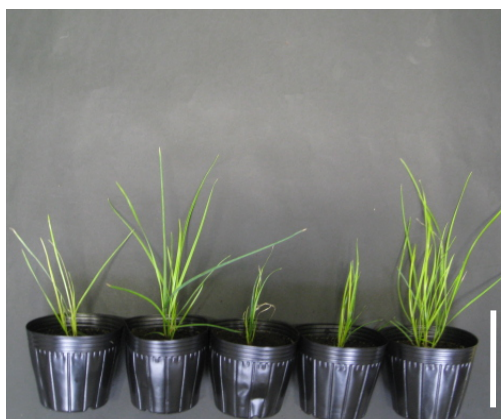

**$\Delta cdc42$**

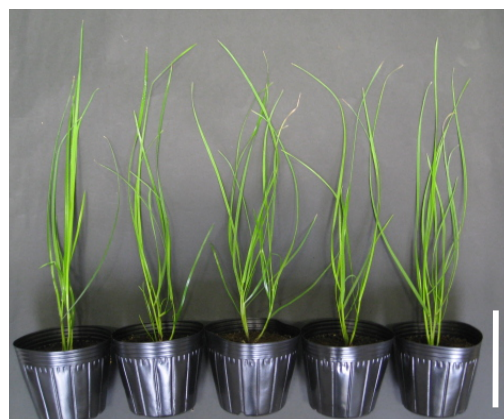

Supplement: S9 Fig — Photographs were taken approx. 2 months after inoculation. Bars = 10 cm. (PDF) [file ppat.1006840.s009.pdf]

**A**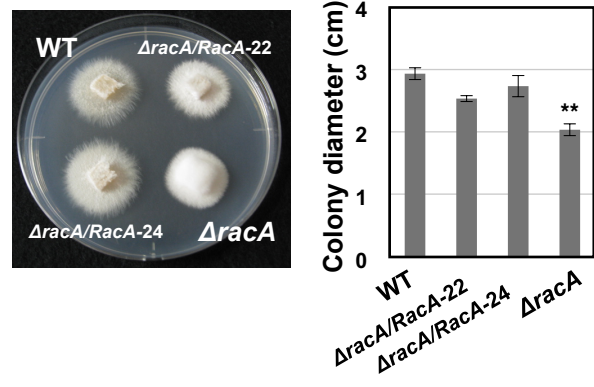**B**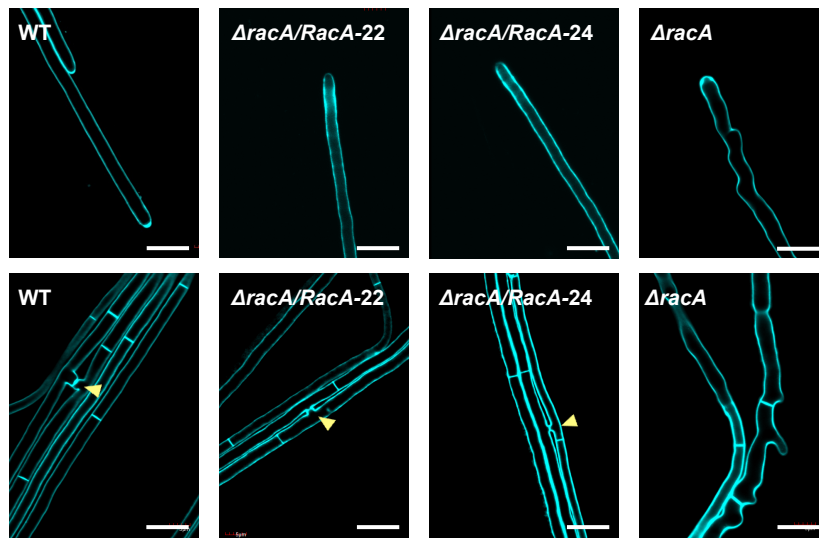**C**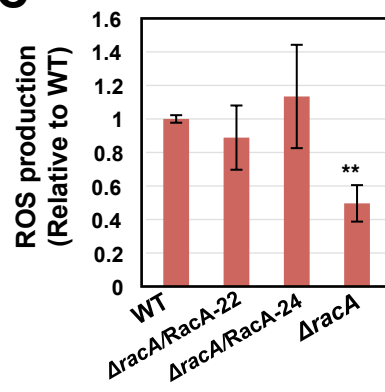**D**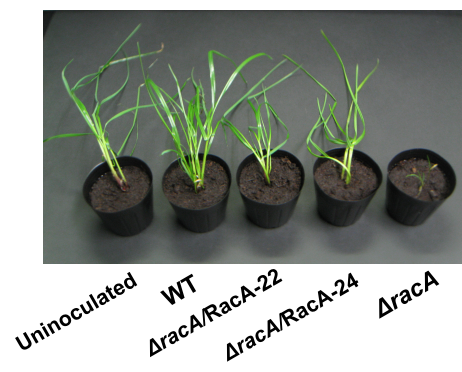

Supplement: S13 Fig — (A) Colony morphology and diameter of E. festucae wild type (WT), racA mutant and complemented strains grown on PDA for 12 days. Data are means ± standard error. n = 3. Data marked with asterisks are significantly different from wild type as assessed by two-tailed Student’s t tests: **P < 0.01. (B) Hyphal growth of WT, racA mutant and complemented strains on water agar. E. festucae strains were stained with calcofluor white and monitored with confocal laser microscopy. Arrowheads indicate hyphal fusions. Bars = 10 μm. (C) L-012-mediated detection of ROS production by E. festucae WT, racA mutant and complemented strains. Colony edge of endophyte strains grown on PDA was treated with L-012 and ROS production was detected as chemiluminescence. Value of chemiluminescence relative to wild type was scored. Data are means ± standard error. n = 5. Data marked with asterisks are significantly different from wild type as assessed by two-tailed Student’s t tests: **P < 0.01. (D) Phenotype of perennial ryegrass infected with E. festucae WT, racA mutant or complemented strains. Photographs were taken approx. 8 weeks after inoculation. (PDF) [file ppat.1006840.s013.pdf]

**A**

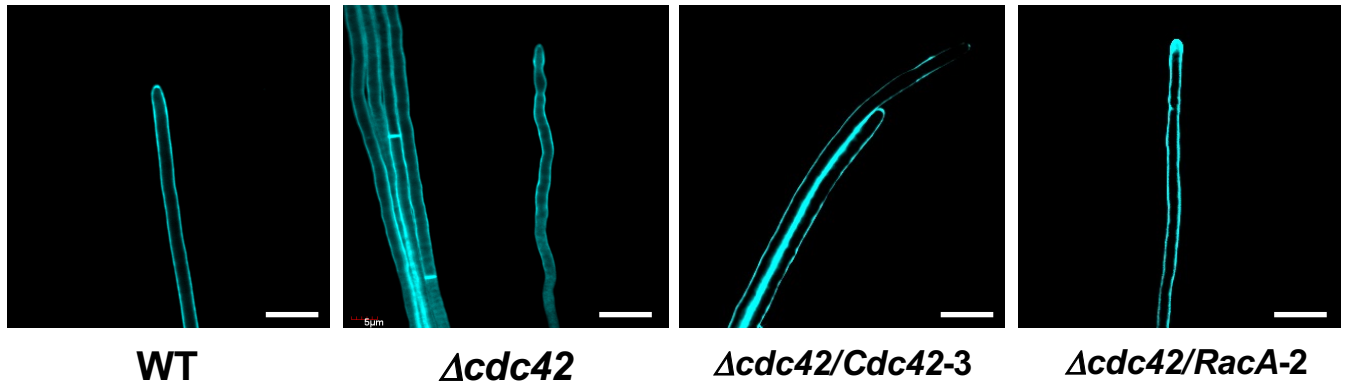

**B**

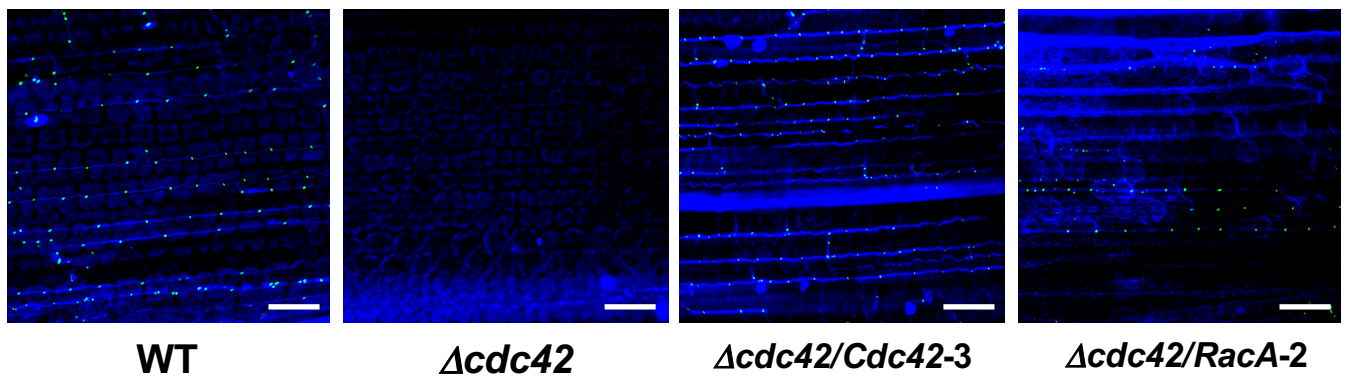

Supplement: S14 Fig — (A) E. festucae strains were grown on 3%water agar for 10 days. Hyphae of endophyte strains were stained with calcofluor white and monitored with confocal laser microscopy. Bars = 30 μm. (B) Colonization of E. festucae WT, cdc42 mutant and complemented strains in top part of perennial ryegrass tillers approx. 2 months after inoculation. Hyphae (blue lines) and septa (green dots) were visualized by WG-AF488/aniline blue staining monitored by confocal microscopy. Bars = 40 μm. (PDF) [file ppat.1006840.s014.pdf]
