# Supplementary material for: Two closely related Rho GTPases, Cdc42 and RacA, of the en-dophytic fungus Epichloë festucae have contrasting roles for ROS production and symbiotic infection synchronized with the host plant
Source: PLoS Pathog. 2018 Jan 25;14(1):e1006840. doi: 10.1371/journal.ppat.1006840 (PMC5785021; doi:10.1371/journal.ppat.1006840)

Ef-Cdc42

```
Sequence     MAVVATIKCVVVGDGAVGKTCLLISYTTNKFPSEYVPTVFDNYAVTVMIGDEPYTLGLFDTAGQEDYDRLRPLSYPQTDVFLVCFSVTSPASFENVREKW
Prediction   CCCCCSSSSSSSCCCCCCHHHHHHHHHHCCCCCCCCCCCCCSSSSSSSSCCSSSSSSSSSCCCCCCCCCHHHHHCCCCCSSSSSSSCCCHHHHHHHHHHH
Conf.Score   99763578999999976799999999972989974588774115889999999999999887899753200145436799899999989976779999999
Sol. Acces.  7542441000000120000000001010333135511000011122334166440300000003243144113111440100000000233601610473 0

             FPEVHHHCPGVPCLIVGTQVDLREDPSVKDKLAKQKMAPVKKEDGDRMARELGAVKYVECSALTQFRLKDVFDEAIVAALEPPMPKKKSHKCLVL
             HHHHHHCCCCCCSSSSSSCCCCCCCHHHHHHHHHCCCCCCCHHHHHHHHHHHCCCSSSSCCCCCCCCHHHHHHHHHHHHHHCCCCCCCCCCCSSC
             99998538899889998894023354668887645688779999999999980998899788687979799999999999707667889998 8759
             24103620470000000013002636622540474644204373034005404032000000234630440032002100335454657434301
```

H:Helix; S:Strand; C:Coil

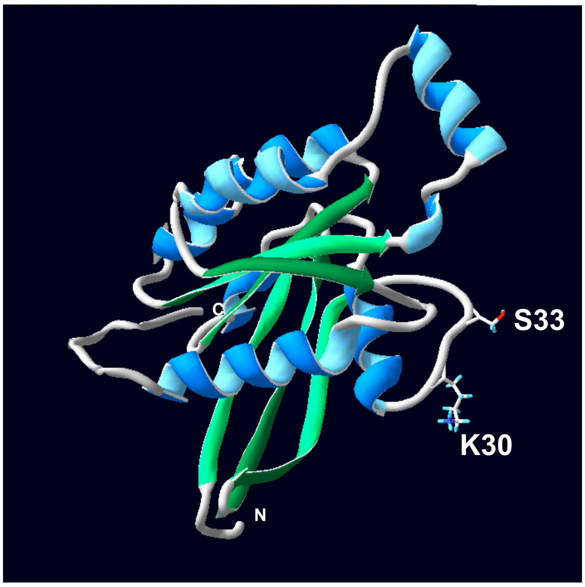

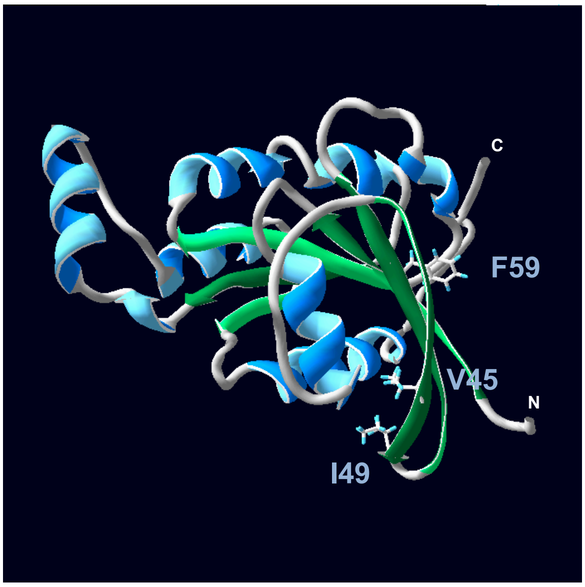

Ef-RacA

```
Sequence     MAQPGVQSLKCVVTGDGAVGKTCLLISYTTNAFPGEYIPTVFDNYSASVMVDGKPISLGLWDTAGQEDYDRLRPLSYPQTDVFLICFSIVSPPSFDNVKA
Prediction   CCCCCCSSSSSSSSCCCCCCHHHHHHHHHCCCCCCCCCCCCSSSSSSSSSSCCSSSSSSSSSCCCCCHHCCCHHHHCCCCCSSSSSSSCCCHHHHHHHHH
Conf.Score   9898764479999999998789999999996098999768971377889999999999999887888321300125434899899999989888899999 99
Sol. Acces.  8556414300000000200000000100145514541100011222030315644030000000314314411311145010000000013360162037

             KWYPEIDHHAPNIPIILVGTKLDLREDAATLDSLRQKRMEPVSYEQALACAREIKAYKYLECSALTQRNLKSVFDEAIRAVLNPRPQPSKHKKSKCSIL
             HHHHHHHHCCCCCCSSSSSSCCCCCCCHHHHHHHHHCCCCCCCHHHHHHHHHHHCCCSSSSCCCCCCCCHHHHHHHHHHHHHHHCCCCCCCCCCCCSSC
             999999972999998999789854455566777865568843389999999998099789989888786979999999999998527555688999888 79
             202410362147000000001200263762264047474520437303400640403210000023463044003300310033575654655441406
```

H:Helix; S:Strand; C:Coil

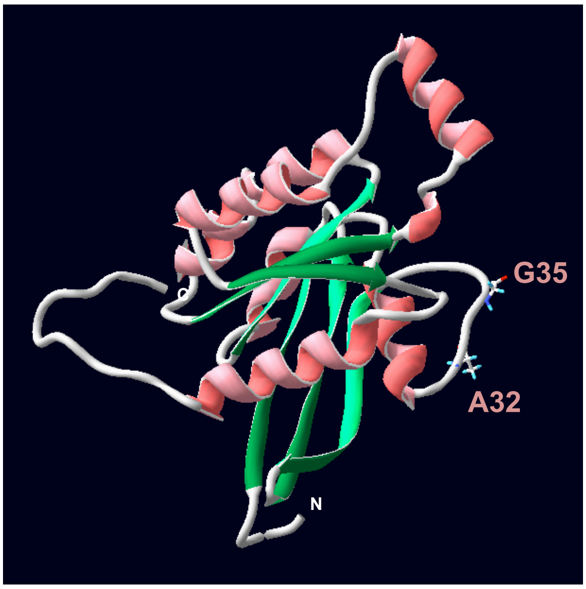

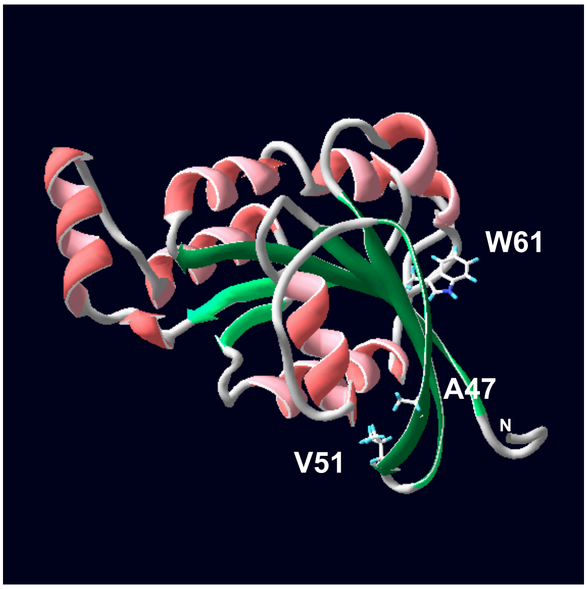

Supplement: S10 Fig — Three-dimensional structure of Rho GTPases are predicted by iterative threading assembly refinement (I-TASSER [61]). Amino acid residues required for specific binding between Cdc42 and BemA, or RacA and NoxR are indicated blue and red letters, respectively. Conf. Score; Confidence score, Sol. Acces.; Predicted solvent accessibility. (PDF) [file ppat.1006840.s010.pdf]
