# Supplementary material for: Two closely related Rho GTPases, Cdc42 and RacA, of the en-dophytic fungus Epichloë festucae have contrasting roles for ROS production and symbiotic infection synchronized with the host plant
Source: PLoS Pathog. 2018 Jan 25;14(1):e1006840. doi: 10.1371/journal.ppat.1006840 (PMC5785021; doi:10.1371/journal.ppat.1006840)
Supplement: S12 Fig — (A) Yeast two-hybrid assays of the interactions between E. festucae NoxR and mutated RacA. (B) Yeast two-hybrid assays of the interactions between E. festucae BemA and chimeric or mutated Cdc42 and RacA. Rho GTPases have mutation in C-terminal plasma membrane localization signal. Yeast strain AH109 was transformed with prey and baid vector as indicated and plated on to SD medium lacking leucine and tryptophan (-L/-T) or lacking leucine, tryptophan, histidine and adenine (-L/-T/-H/-A). Growth on the latter indicates an interaction between bait and prey. (PDF) [file ppat.1006840.s012.pdf]

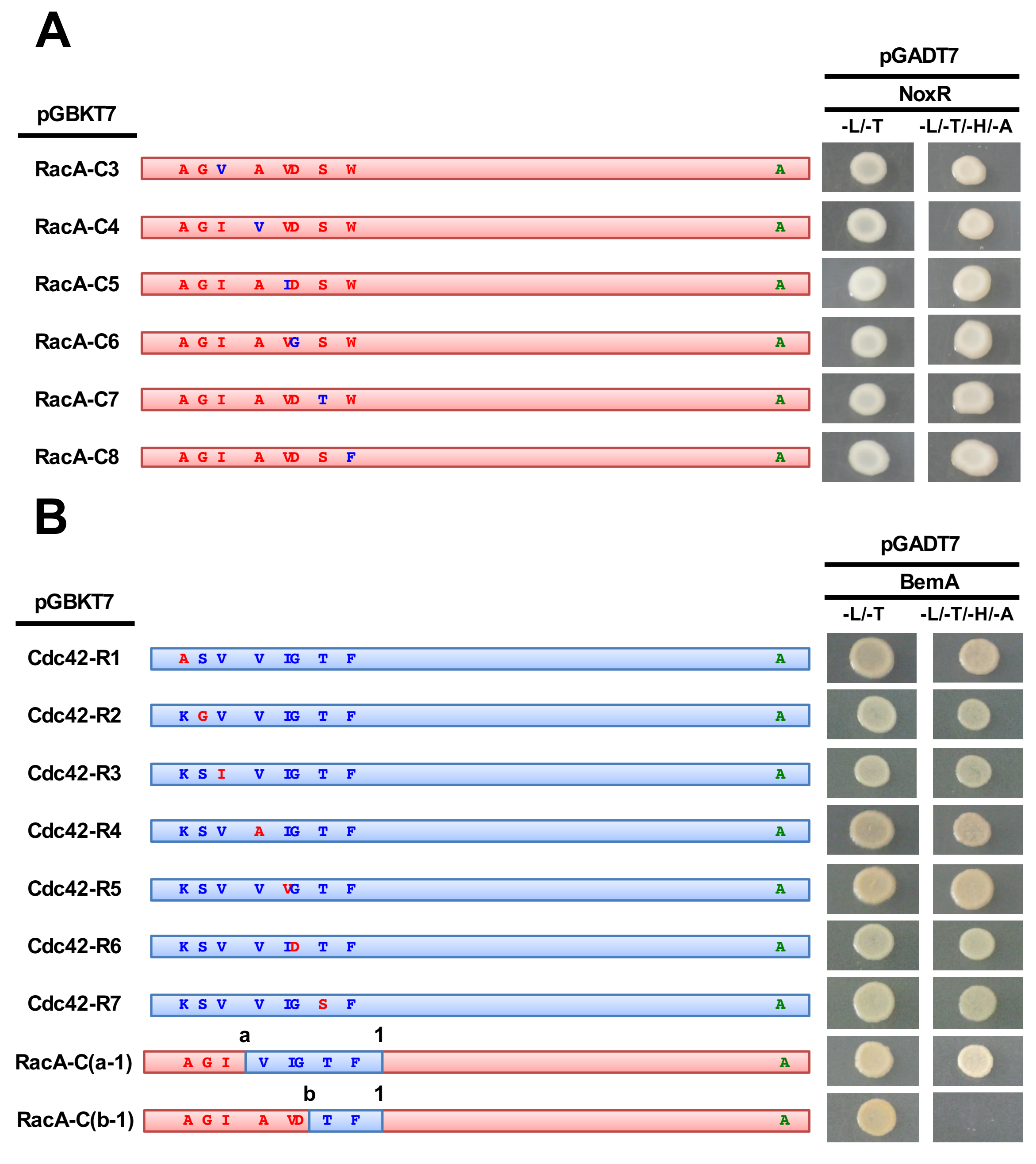
A
B
pGBKT7
pGADT7
NoxR
BemA
-L/-T
-L/-T/-H/-A
RacA-C3
RacA-C4
RacA-C5
RacA-C6
RacA-C7
RacA-C8
Cdc42-R1
Cdc42-R2
Cdc42-R3
Cdc42-R4
Cdc42-R5
Cdc42-R6
Cdc42-R7
RacA-C(a-1)
RacA-C(b-1)
a
b
1
