# Supplementary material for: Two closely related Rho GTPases, Cdc42 and RacA, of the en-dophytic fungus Epichloë festucae have contrasting roles for ROS production and symbiotic infection synchronized with the host plant
Source: PLoS Pathog. 2018 Jan 25;14(1):e1006840. doi: 10.1371/journal.ppat.1006840 (PMC5785021; doi:10.1371/journal.ppat.1006840)
Supplement: S11 Fig — Amino acids specifically conserved among fungal Cdc42 and RacA are indicated by blue and red letters, respectively. An; Aspergillus nidulans (strain FGSC A4), Bc; Botrytis cinerea (T4), Bs; Bipolaris sorokiniana (ND90Pr), Co; Colletotrichum orbiculare (MAFF 240422), Cp; Claviceps purpurea (20.1), Ct; Colletotrichum trifolii (race 1), Fg; Fusarium graminearum (PH-1), Fo; Fusarium oxysporum f. sp. cubense (race 1), Mg; Magnaporthe grisea (70–15), Nc; Neurospora crassa (OR74A), Pd; Penicillium digitatum (PHI26), Sr; Sporisorium reilianum (SRZ2), Tm; Talaromyces marneffei (ATCC 18224), Um; Ustilago maydis (Bub8). (PDF) [file ppat.1006840.s011.pdf]

```
EfCdc42   1:   MAVVATIKCVVVGDGAVGKTCLLISYTTNKFPSEYVPTVFDNYAVTVMIGDEPYTLGLFDTAGQEDYDRLRPLSYPQTDVFLVCFSVTSPASFENVREK: 99
AnCdc42   1:    MVVATIKCVVVGDGAVGKTCLLISYTTNKFPSEYVPTVFDNYAVTVMIGDEPYTLGLFDTAGQEDYDRLRPLSYPQTDVFLVCFSVTSPASFENVREK: 98
BcCdc42   1:    MVVATIKCVVVGDGAVGKTCLLISYTTNKFPSEYVPTVFDNYAVTVMIGDEPYTLGLFDTAGQEDYDRLRPLSYPQTDVFLVCFSVTSPASFENVREK: 98
BsCdc42   1:    MVVATIKCVVVGDGAVGKTCLLISYTTNKFPSEYVPTVFDNYAVTVMIGDEPYTLGLFDTAGQEDYDRLRPLSYPQTDVFLVCFSVTSPASFENVREK: 98
CoCdc42   1:    MVVATIKCVVVGDGAVGKTCLLISYTTNKFPSEYVPTVFDNYAVTVMIGDEPYTLGLFDTAGQEDYDRLRPLSYPQTDVFLVCFSVTSPASFENVREK: 98
CpCdc42   1:   MAVVATIKCVVVGDGAVGKTCLLISYTTNKFPSEYVPTVFDNYAVTVMIGDEPYTLGLFDTAGQEDYDRLRPLSYPQTDVFLVCFSVTSPASFENVREK: 99
CtCdc42   1:    MVVATIKCVVVGDGAVGKTCLLISYTTNKFPSEYVPTVFDNYAVTVMIGDEPYTLGLFDTAGQEDYDRLRPLSYPQTDVFLVCFSVTSPASFENVREK: 98
FgCdc42   1:   MAVVATIKCVVVGDGAVGKTCLLISYTTNKFPSEYVPTVFDNYAVTVMIGDEPYTLGLFDTAGQEDYDRLRPLSYPQTDVFLVCFSVTSPASFENVREK: 99
FoCdc42   1:   MAVVATIKCVVVGDGAVGKTCLLISYTTNKFPSEYVPTVFDNYAVTVMIGDEPYTLGLFDTAGQEDYDRLRPLSYPQTDVFLVCFSVTSPASFENVREK: 99
MgCdc42   1:    MVVATIKCVVVGDGAVGKTCLLISYTTNKFPSEYVPTVFDNYAVTVMIGDEPYTLGLFDTAGQEDYDRLRPLSYPQTDVFLVCFSVTSPASFENVREK: 98
NcCdc42   1:    MVTGTIKCVVVGDGAVGKTCLLISYTTNKFPSEYVPTVFDNYAVTVMIGDEPYTLGLFDTAGQEDYDRLRPLSYPQTDVFLICFSVASPASFENVSQK: 98
PdCdc42   1:    MVVATIKCVVVGDGAVGKTCLLISYTTNKFPSEYVPTVFDNYAVTVMIGDEPYTLGLFDTAGQEDYDRLRPLSYPQTDVFLVCFSVTSPASFENVREK: 98
SrCdc42   1:      MQTIKCVVVGDGAVGKTCLLISYTTNKFPSEYVPTVFDNYAVTVMIGEDPYTLGLFDTAGQEDYDRLRPLSYPQTDVFLVCFSVTSPASFENVKEK: 96
TmCdc42   1:    MVVATIKCVVVGDGAVGKTCLLISYTTNKFPSEYVPTVFDNYAVTVMIGDEPYTLGLFDTAGQEDYDRLRPLSYPQTDVFLVCFSVTSPASFENVREK: 98
UmCdc42   1:      MQTIKCVVVGDGAVGKTCLLISYTTNKFPSEYVPTVFDNYAVTVMIGEDPYTLGLFDTAGQEDYDRLRPLSYPQTDVFLVCFSVTSPASFENVKEK: 96
EfRacA    1:MAQ-PGVQSLKCVVTGDGAVGKTCLLISYTTNAFPGEYIPTVFDNYSASVMVDGKPISLGLWDTAGQEDYDRLRPLSYPQTDVFLICFSIVSPPSFDNVKAK:101
AnRac     1:MATGPATQSLKCVVTGDGAVGKTCLLISYTTNAFPGEYIPTVFDNYTASVMVDGRPISLGLWDTAGQEDYDRLRPLSYPQTDVFLICFSIVSPPSFDNVKSK:102
BcRac     1:MAA-PGTQSLKCVVTGDGAVGKTCLLISYTTNAFPGEYIPTVFDNYSASVMVDGKPISLGLWDTAGQEDYDRLRPLSYPQTDVFLICFSIVSPPSFDNVRAK:101
BsRac     1:MAA-PATQSLKCVVTGDGAVGKTCLLISYTTNAFPGEYIPTVFDNYSANVMVDGKPISLGLWDTAGQEDYDRLRPLSYPQTDVFLICFSIVSPPSFDNVKAK:101
CoRac     1:MAQ-PGVQSLKCVVTGDGAVGKTCLLISYTTNAFPGEYIPTVFDNYSASVMVDGKPISLGLWDTAGQEDYDRLRPLSYPQTDVFLICFSIVSPPSFDNVKAK:101
CpRac     1:MAQ-PGVQSLKCVVTGDGAVGKTCLLISYTTNAFPGEYIPTVFDNYSASVMVDGKPISLGLWDTAGQEDYDRLRPLSYPQTDVFLICFSIVSPPSFDNVKAK:101
CtRac     1:MAQ-PGVQSLKCVVTGDGAVGKTCLLISYTTNAFPGEYIPTVFDNYSASVMVDGKPISLGLWDTAGQEDYDRLRPLSYPQTDVFLICFSIVSPPSFDNVKAK:101
FgRac     1:MAQ-PGVQSLKCVVTGDGAVGKTCLLISYTTNAFPGEYIPTVFDNYSASVMVDGKPISLGLWDTAGQEDYDRLRPLSYPQTDVFLICFSIVSPPSFDNVKAK:101
FoRac     1:MAQ-PGVQSLKCVVTGDGAVGKTCLLISYTTNAFPGEYIPTVFDNYSASVMVDGKPISLGLWDTAGQEDYDRLRPLSYPQTDVFLICFSIVSPPSFDNVKAK:101
MgRac     1:MAA-PGVQSLKCVVTGDGAVGKTCLLISYTTNAFPGEYIPTVFDNYSASVMVDGKPISLGLWDTAGQEDYDRLRPLSYPQTDVFLICFSIVSPPSFDNVKAK:101
NcRac     1:MAAIGGVQSIKCVVTGDGAVGKTCLLISYTTNAFPGEYIPTVFDNYSASVMVDGKPVSLGLWDTAGQEDYDRLRPLSYPQTDVFLICFSLVSPPSFDNVKSK:102
PdRac     1:MATGPATQSLKCVVTGDGAVGKTCLLISYTTNAFPGEYIPTVFDNYTASVMVDGRPISLGLWDTAGQEDYDRLRPLSYPQTDVFLICFSIVSPPSFDNVKAK:102
SrRac     1:      MQTIKCVVVGDGAVGKTCLLISYTTNAFPGEYIPTVFDNYSANVMVDGKPVSLGLWDTAGQEDYDRLRPLSYPQTDVFLICFSLVSPPSFENVRTK: 96
TmRac     1:MAQ-PGVQSLKCVVTGDGAVGKTCLLISYTTNAFPGEYIPTVFDNYSASVMVDGKPISLGLWDTAGQEDYDRLRPLSYPQTDVFLICFSIVSPPSFDNVKAK:101
UmRac     1:      MQTIKCVVVGDGAVGKTCLLISYTTNAFPGEYIPTVFDNYSANVMVDGKPVSLGLWDTAGQEDYDRLRPLSYPQTDVFLVCFSLVSPPSFENVRTK: 96

EfCdc42 100:WFPEVHHHCPGVPCLIVGTQVDLREDPSVKDKLAKQKMAPVKKEDGDRMARELGAVKYVECSALTQFRLKDVFDEAIVAALEPPMPKK-------KSHKCLVL:195
AnCdc42  99:WFPEVHHHCPGVPCLIVGTQVDLRDDPAVRDKLARQKMQPIRKEDGDRMAKDLGAVKYVECSALTQYKLKDVFDEAIVAALEP-APKK-------RSR-CVLL:192
BcCdc42  99:WFPEVHHHCPGVPCLIVGTQTDLRDDPSVKEKLTKQKMKPVEKSDGERMAKDLGAVKYVECSALTQYKLKDVFDEAIVAALEPPAPKK-------KQHKCLIL:194
BsCdc42  99:WFPEVHHHCPGVPCLIVGTQVDLREDASVKDKLSKQRMAPVKKEDGERMARELGAVKYVECSALTQFKLKDVFDEAIVAALEPPATKKEGGERKKGKKCSIL:200
CoCdc42  99:WFPEVHHHCPGVPCLIVGTQVDLREDSSVREKLSKQKMSPVRKEDGERMAKDLGAVKYVECSALTQYKLKDVFDEAIVAALEPPAPKK-------KSHKCLVL:194
CpCdc42 100:WFPEVHHHCPGVPCLIVGTQVDLRDDPSVREKLAKQKMAPVKKEEGDKMARELGAVKYVECSALTQFRLKDVFDEAIVAALEPPMPKK-------KSLKCLIL:195
CtCdc42  99:WFPEVHHHCPGVPCLIVGTQVDLREDPSVREKLSKQKMSPVRKEDGERMAKDLGAVKYVECSALTQYKLKDVFDEAIVAALEPPAPKK-------KSHKCLVL:194
FgCdc42 100:WFPEVRHHCPGVPCLIVGTQVDLRDDPSVREKLSKQKMQPVRREDGERMAKDLGAVKYVECSALTQYKLKDVFDEAIVAALEPPAPKK-------KSHKCLVL:195
FoCdc42 100:WFPEVRHHCPGVPCLIVGTQVDLRDDPSVREKLSKQKMQPVRREDGERMAKDLGAVKYVECSALTQYKLKDVFDEAIVAALEPPAPKK-------KSHKCLVL:195
MgCdc42  99:WFPEVHHHCPGVPCLIVGTQVDLRDDPSVREKLSKQKMQPVRREDGERMAKELGAVKYVECSALTQYKLKDVFDEAIVAALEPPTPKR-------KSKKCLIL:194
NcCdc42  99:WAPEVNHHCPGVPFLIVGTQKDLRSDKELRDKLAQRKQSMIEFKQGEKLAQDLDAVKYVECSALTQEGLKNVFDEAIVAALEPPQKKTS----KRDKKCLIL:196
PdCdc42  99:WFPEVHHHCPGVPCLIVGTQTDLRDDAGVRDKLARQKMSPICKEDGDRMAKELGAVKYVECSALTQYKLKDVFDEAIVAALEP-APKK-------KSKGCLLL:193
SrCdc42  97:WFPEVHHHCPGVPCLIVGTQVDLRDDQAVIEKLARSKQRPVPFEGGERLARELGAVKYVECSALTQKGLKNVFDEAIVAALEPPVIRK-------KSK-CAIL:191
TmCdc42  99:WFPEVHHHCPGVPCLIVGTQTDLRDDPAVREKLAKQKMQPVRKEDGDRMAKELGAVKYVECSALTQYKLKDVFDEAIVAALEP-APKK-------RPK-CVVL:192
UmCdc42  97:WFPEVHHHCPGVPCLIVGTQVDLRDDHAVIEKLARSKQRPVPFEAGERLARELGAVKYVECSALTQKGLKNVFDEAIVAALEPPVIRK-------KSK-CAIL:191
EfRacA  102:WYPEIDHHAPGVPIILVGTKLDLREDAATLDSLRQKRMEPVSYEQALACAREIKAYKYLECSALTQRNLKSVFDEAIRAVLNPRPQPSKHK---KSK-CSIL:199
AnRac   103:WFPEIEHHAPGVPIILVGTKLDLRDDPAQLESLRMRKQEPVTYEQALAVAKEIRAHKYLECSALTQRNLKSVFDEAIRAVLNPRPAT --QR--NKK-CTIL:199
BcRac   102:WFPEISHHAPGVPIILVGTKLDLRDDEPTKESLRSKRMEPVTYDQARVVAKEIRAHKYLECSALTQRNLKSVFDEAIRAVLSPQQQAPKPK---KSK-CTVL:199
BsRac   102:WYPEIDHHAPGVPIILVGTKLDLRDDEATKESLRQKKMAPIQYEQAVMVAKEIKAQKYLECSALTQRNLKSVFDEAIRAVLSPRPQVSAAKN--KKK-CTIL:200
CoRac   102:WYPEIDHHAPNIPIILVGTKLDLREDPNTLESLRQKRMEPVSYDQALICAKEIKAHKYLECSALTQRNLKSVFDEAIRAVLNPRPVAQQKK---KSK-CTIL:199
CpRac   102:WHPEIDHHAPNIPIILVGTKLDLREDAATLDSLRQKRMEPVSYEQALACAREIKACKYLECSALTQRNLKSVFDEAIRAVLNPRSQTSKSK---KSK-CSIL:199
CtRac   102:WYPEIDHHAPNIPIILVGTKLDLREDPNTLESLRQKRMEPVSYDQALICAKEIKAHKYLECSALTQRNLKSVFDEAIRAVLNPRPVAQQKK---KSK-CTIL:199
FgRac   102:WYPEIDHHAPNIPIILVGTKLDLREDASTLESLRQKRMEPVSYEQALTCAKEIKAYKYLECSALTQRNLKSVFDEAIRAVLNPRPTPSKQK---KNK-CSIL:199
FoRac   102:WYPEIDHHAPNIPIILVGTKLDLREDASTLESLRQKRMEPVSYEQALTCAKEIKAYKYLECSALTQRNLKSVFDEAIRAVLNPRPTPSKQK---KNK-CSIL:199
MgRac   102:WYPEIDHHAPNVPIILVGTKLDLREDPSTLESLRSKRMEPVSYDQALICAKEIRAHKYLECSALTQRNLKSVFDEAIRAVLNPRPQPAKVK---KSK-CTIL:199
NcRac   103:WHPEIQHHAPGIPIILVGTKLDLREDPDTIQSLSQKRMAPITFEMGVNCAKEIGARKYLECSALTQRNLKSVFDEAIRAVLYRVDTTQEKK---KSK-CTIL:200
PdRac   103:WFPEIEHHAPNVPIILVGTKLDLRDDPATTQALHARKMETVSYEQALAVAKEIRAHKYLECSALTQRNLKSVFDEAIRAVLNPRPTTKSGRK--AAK-CNIL:201
SrRac    97:WWPEISHHAPNIPTILVGTKLDLREDPETIAKLRDRRMQPITYAQGNQMARDIHANKYLECSALTQKGLKGVFDEAIRSVLAPAP-VKSKK---KNN-CTIL:193
TmRac   102:WYPEIDHHAPNIPIILVGTKLDLREDASTLESLRQKRMEPVSYEQALTCAKEIKAYKYLECSALTQRNLKSVFDEAIRAVLNPRPTPSKQK---KNK-CSIL:199
UmRac    97:WWPEVSHHAPNIPTILVGTKLDLREDPETIAKLRDRRMQPITYAQGNQMARDIHATKYLECSALTQKGLKGVFDEAIRSVLAPAP-VQSKK---KNN-CLIL:193
```
